# Structural Basis of Sequential Enantioselective Epoxidation by a Flavin‐Dependent Monooxygenase in Lasalocid A Biosynthesis

**DOI:** 10.1002/anie.202504982

**Published:** 2025-04-26

**Authors:** Qian Wang, Yaming Deng, Dayan Viera, Xiaopeng Liu, Ning Liu, Yulu Hu, Xiangdong Hu, Hao Wei, Quan Zhou, Ting Lan, Wei He, Xi Chen, Chu‐Young Kim

**Affiliations:** ^1^ Key Laboratory of Synthetic and Natural Functional Molecule of the Ministry of Education College of Chemistry and Materials Science Northwest University Xi'an 710127 China; ^2^ Department of Biochemistry School of Molecular and Cellular Biology University of Illinois Urbana‐Champaign Urbana Illinois 61801 USA; ^3^ Institute for Genomic Biology University of Illinois Urbana‐Champaign Urbana Illinois 61801 USA; ^4^ Department of Chemistry University of Illinois Urbana‐Champaign Urbana Illinois 61801 USA; ^5^ Department of Chemistry and Biochemistry The University of Texas at El Paso El Paso Texas 79968 USA; ^6^ Department of Chemistry School of Pharmacy Air Force Medical University Xi'an 710032 China

**Keywords:** Epoxidation, Monooxygenase, Polyethers, Polyketides, Protein structures

## Abstract

Polyether polyketides are a structurally diverse group of natural products known for their antimicrobial and antiproliferative activities. Lasalocid A is a canonical natural polyether produced by the soil bacterium *Streptomyces lasalocidi*. In lasalocid A biosynthesis, a polyene polyketide intermediate is converted into a bisepoxide by the flavin‐dependent monooxygenase enzyme Lsd18. Remarkably, Lsd18 acts on two distinct C═C groups in the substrate molecule, forming two (*R*,*R*) epoxides. We have determined the X‐ray crystal structures of Lsd18 in the substrate‐free, substrate‐bound, and product‐bound forms. Our work has revealed that Lsd18 has an extra‐large substrate‐binding pocket that allows the polyene to adopt different conformations within the enzyme pocket. This feature enables Lsd18 to epoxidate both of the C═C groups. Additionally, a subpocket located near the Lsd18 active site controls stereoselectivity by dictating which face of the C═C group is placed next to the flavin. Molecular understanding of how Lsd18 transforms a polyene into a bisepoxide during lasalocid A biosynthesis lays the foundation for the production of designer polyethers for drug development.

## Graphical abstract

Lsd18 is a flavin‐dependent monooxygenase from *Streptomyces lasalocidi* that performs two enantioselective epoxidations during lasalocid A biosynthesis. X‐ray crystal structures of Lsd18 bound to a substrate and product analogue illuminate how this enzyme performs multiple epoxidations on the same substrate molecule and how it controls stereoselectivity.

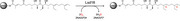

## Introduction

Polyethers are a subgroup of polyketide natural products, and they are characterized by the presence of multiple cyclic ethers in their structure. Examples of natural polyethers include lasalocid A, monensin, nanchangmycin, nigericin, and salinomycin. Nowadays, polyethers are primarily used as anticoccidial agents and as growth promoters in farm animals.^[^ ^1^, ^2^ ^]^ Although their precise mechanism has not been determined, polyethers are known to bind specific cations and render them permeable to biological membranes, thereby disrupting ion homeostasis.^[^ ^3^ ^]^ Polyethers are currently not used as human therapeutics because of toxicity issues.^[^ ^4^, ^5^ ^]^ This limitation could be overcome by modifying the structure of natural polyethers.^[^ ^6^, ^7^, ^8^ ^]^ However, polyethers are challenging to synthesize chemically due to the large number of stereocenters they contain. Our goal is to achieve a detailed understanding of how microorganisms synthesize natural polyethers to enable bioproduction of polyether analogues in the laboratory and thereby accelerate polyether drug development.

Lasalocid A is a prototypical polyether. It is a dodecaketide containing two cyclic ethers and 10 stereocenters. Lasalocid A was first discovered in 1951 by researchers at Hoffmann‐La Roche through screening soil samples for antimicrobial activity.^[^ ^9^ ^]^ In 1974, Westley and colleagues determined that the backbone of lasalocid A is derived from five acetate, four propionate, and three butyrate.^[^ ^10^ ^]^ Total synthesis of lasalocid A was first achieved in 1978 by the Kishi group, and since then additional total syntheses have been reported.^[^ ^11^, ^12^, ^13^ ^]^ In 2008, the Leadlay group cloned and sequenced the lasalocid A biosynthetic gene cluster from *Streptomyces lasalocidi* (ATCC 31180).^[^ ^14^ ^]^ This gene cluster contains putative genes for seven type I modular polyketide synthases (PKSs) (*lasAI‐lasAVII*), one monooxygenase (*lasC*), and one epoxide hydrolase (*lasB*). In 2009, the Oikawa group reported the lasalocid A biosynthetic gene cluster from *S. lasalocidi* (ATCC 35851).^[^ ^15^ ^]^ Like the Leadlay group, they identified putative genes for seven type I modular PKSs (*lsd11‐lsd17*), one monooxygenase (*lsd18*), and one epoxide hydrolase (*lsd19*). The Las and Lsd enzymes are structurally and functionally equivalent. In 2012, our group solved the X‐ray crystal structure of the Lsd19 epoxide hydrolase containing a substrate analogue and a product analogue.^[^ ^16^ ^]^ In 2015, we determined the X‐ray crystal structure of Lsd19 in complex with lasalocid A.^[^ ^17^ ^]^ In 2021, we reported the X‐ray crystal and cryo‐EM structures of the Lsd14 PKS.^[^ ^18^ ^]^

**Scheme 1:**
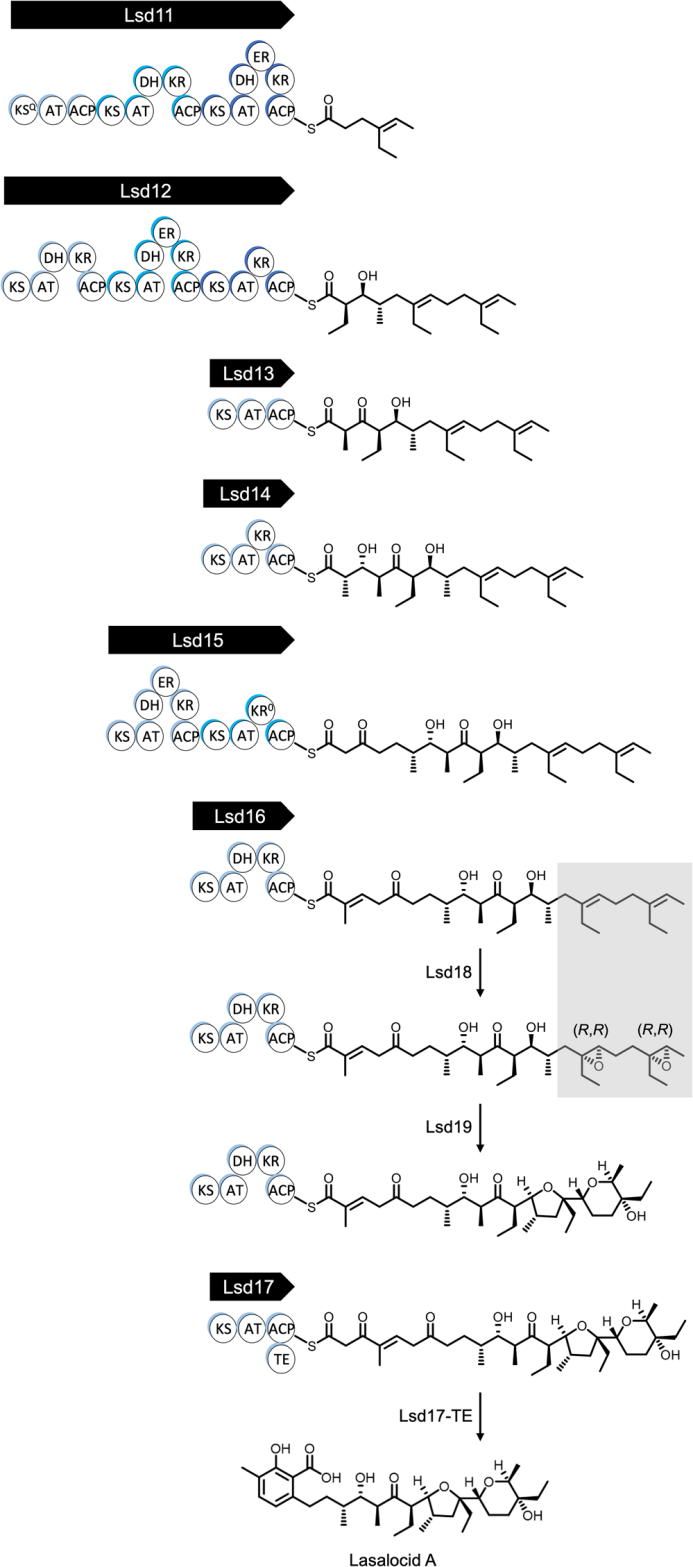
Predicted lasalocid A biosynthesis pathway in *Streptomyces lasalocidi*. Lsd11 to Lsd17 are type I modular polyketide synthases. (ACP = acyl carrier protein, AT = acyltransferase, DH = dehydratase, ER = enoylreductase, KS = ketosynthase, KS*^Q^* = KS‐like decarboxylase, KR = ketoreductase, KR^0^ = inactive KR, TE = thioesterase). Lsd18 is a flavin‐dependent monooxygenase, and Lsd19 is an epoxide hydrolase. The growing polyketide chain is covalently tethered to the ACP domain via a thioester linkage. The TE domain of Lsd17 is thought to catalyze the polyketide chain release and the formation of the 3‐methylsalicylate moiety. The timing of the epoxidation reaction and cyclic ether formation reaction are not conclusively resolved.

The putative lasalocid A biosynthesis pathway is shown in Scheme 1. First, an all‐*trans* undecaketide polyene intermediate is constructed by six modular PKSs, Lsd11 to Lsd16. Second, two C═C groups in the polyene polyketide are oxygenated by the monooxygenase, Lsd18, to yield a bisepoxide. Third, the iconic cyclic ethers are formed by the epoxide hydrolase, Lsd19. Lastly, the Lsd17 PKS extends the growing chain by two carbon atoms, releases the dodecaketide product, and putatively forms the aromatic 3‐methylsalicylate group. It should be noted that the timing of the Lsd18‐catalyzed epoxidation and the Lsd19‐catalyzed cyclic ether formation are not definitively known. According to a substrate off‐loading study performed by the Leadlay group, the epoxidation likely takes place on either the decaketide tethered to LasAV (Lsd15) or the undecaketide tethered to LasAVI (Lsd16).^[^ ^19^ ^]^

The main objective of this study is to elucidate how Lsd18 performs two epoxidation reactions on both the internal and terminal C═C group in the polyene precursor and how it selectively generates (*R*,*R*) epoxides. The stereochemistry of the Lsd18‐catalyzed epoxidation reaction is of particular interest as it determines the fate of six out of ten chiral centers in lasalocid A. To determine the molecular basis of the stereoselectivity of Lsd18, we have determined three X‐ray crystal structures. 1) Lsd18 in complex with the coenzyme flavin adenine dinucleotide (FAD), 2) Lsd18 in complex with FAD and a diene substrate analogue, and 3) Lsd18 in complex with FAD and a bisepoxide product analogue. Next, we have conducted a mutagenesis study to further probe the stereocontrol mechanism of Lsd18. Our work has revealed how Lsd18 single‐handedly transforms a diene polyketide substrate into an (*R*,*R*),(*R*,*R*) bisepoxide.

## Results and Discussion

### Catalytic Activity of Lsd18

**Figure 1:**
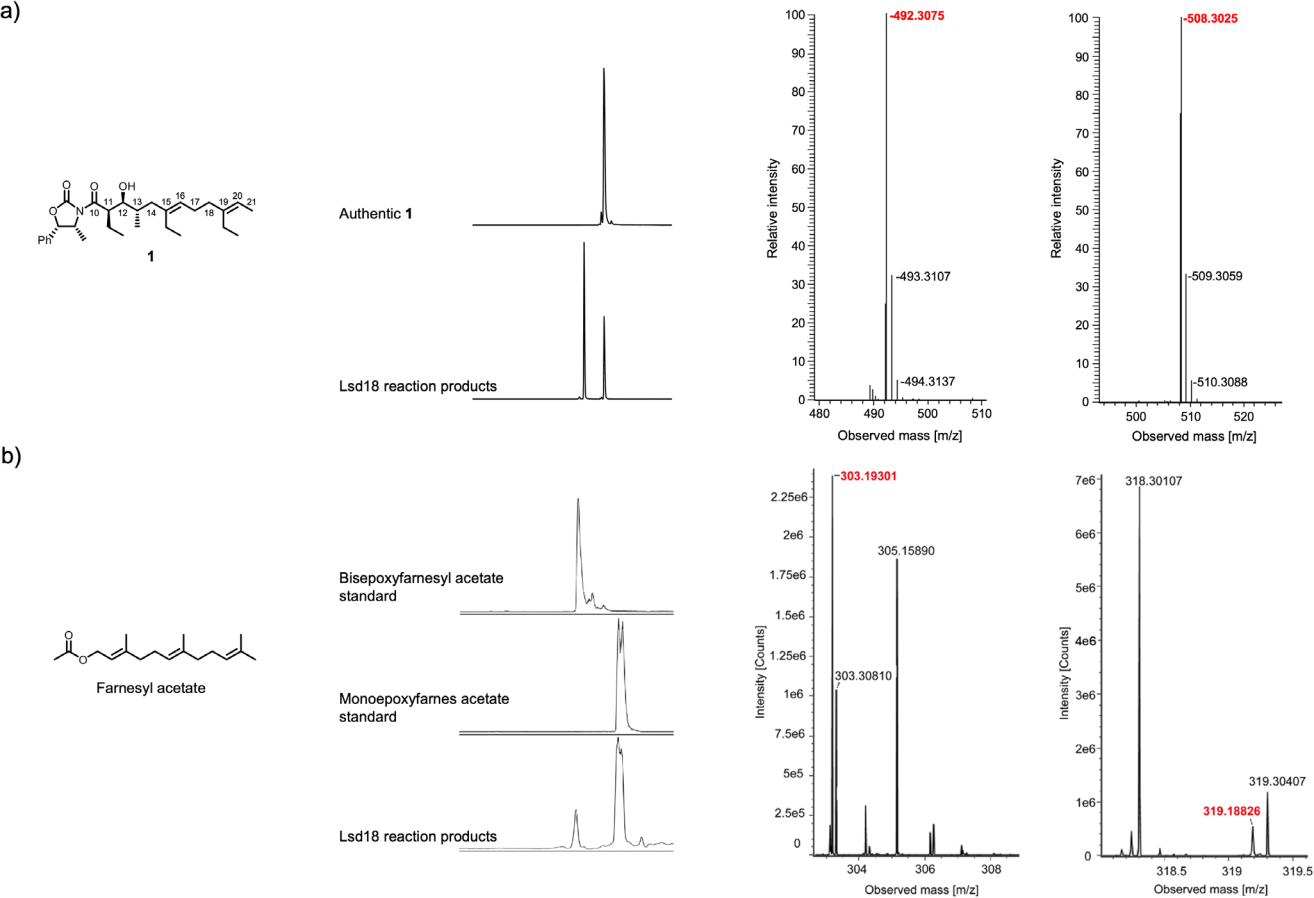
In vitro activity of recombinant Lsd18. a) LC‐MS analysis of **1** before and after incubation with Lsd18. Compound **1** was chemically synthesized. ESI‐MS spectrum corresponding to [M+Na]^+^ of **1** (theoretical mass: 492.3075 Da; observed mass: 492.3075 Da) and [M+Na]^+^ of monoepoxy‐**1** (theoretical mass: 508.3025 Da; observed mass: 508.3025 Da). b) LC‐MS analysis of monoepoxyfarnesyl acetate, bisepoxyfarnesyl acetate, and farnesyl acetate after incubation with Lsd18. The epoxyfarnesyl acetate standards were chemically prepared using commercial farnesyl acetate. ESI‐MS spectrum corresponding to [M+Na]^+^ of monoepoxyfarnesyl acetate (theoretical mass: 303.19301 Da; observed mass: 303.19301 Da) and [M+Na]^+^ of bisepoxyfarnesyl acetate (theoretical mass: 319.18798 Da; observed mass: 319.18826 Da).

The putative native substrate of Lsd18 is an undecaketide that is covalently tethered to a PKS (Scheme 1). Because this substrate is difficult to prepare, we used a diene substrate analogue, **1** (Figure 1a), which is not tethered to a PKS, in our biochemical and crystallographic studies. **1** was chemically synthesized following a published method with modifications.^[^ ^20^ ^]^ Notably, the C11 to C21 section of **1** is structurally identical to the section of the native substrate where the two alkenes that undergo epoxidation are located. Incubation of Lsd18 and **1** along with the required cofactors for 16 h at 30 °C generated a monoepoxide product, indicating that the recombinant Lsd18 protein is catalytically active. However, no bisepoxide product was detected. We reasoned this is because the bulky oxazolidinone head group of **1** prevents the internal alkene (C15═C16) from reaching the enzyme active site. Since we did not detect the expected bisepoxide product with **1**, and because bisepoxide formation by Lsd18 has never been demonstrated in vitro, we tested a variety of alternative polyene substrates. We found that Lsd18 transforms farnesyl acetate, a natural sesquiterpene, into a bisepoxide (Figure 1b). This observation confirms that Lsd18 can perform two epoxidations on the same substrate molecule, which is consistent with its proposed role in lasalocid A biosynthesis.

Flavin‐dependent monooxygenases typically produce the highly reactive C4a‐hydroperoxyflavin species when molecular oxygen reacts with FADH_2_.^[^ ^21^, ^22^, ^23^ ^]^ But in some cases, oxygen reacts with FADH_2_ to form the flavin‐N5‐oxide species.^[^ ^24^, ^25^, ^26^, ^27^, ^28^, ^29^ ^]^ To test the possibility that Lsd18 utilizes the flavin‐N5‐oxide pathway, we measured the ultraviolet‐visible light spectrum of the Lsd18 solution before and 1‐h after incubation with farnesyl acetate under the reaction assay condition mentioned above (Figure S1). In both cases, we did not see an absorbance peak at 460 nm that is characteristic of flavin‐N5‐oxide. Therefore, Lsd18 likely utilizes the C4a‐hydroperoxyflavin pathway.

### X‐Ray Crystal Structure of Lsd18‐FAD

**Figure 2:**
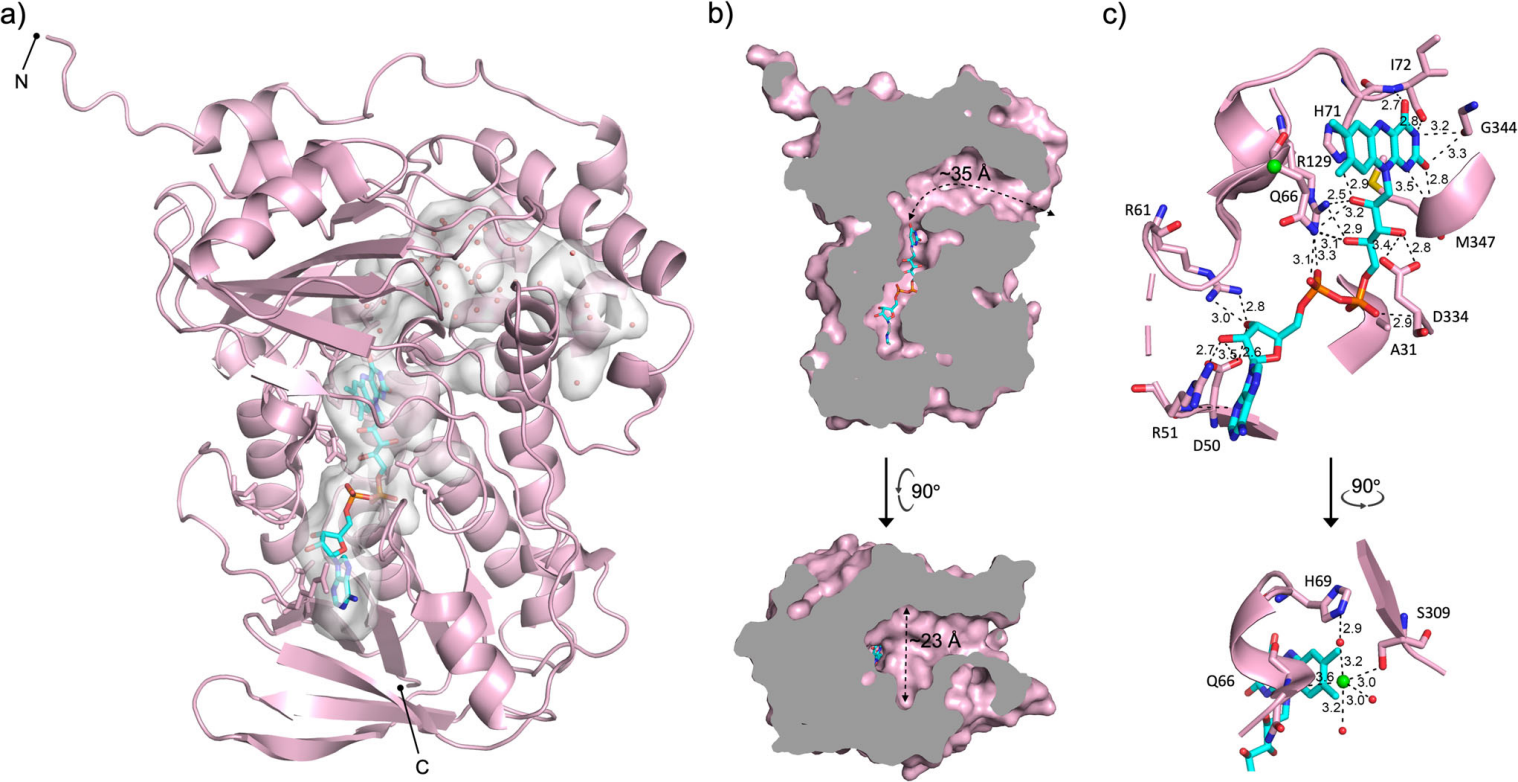
X‐ray crystal structure of Lsd18‐FAD. a) Overall structure of Lsd18. FAD is drawn as a stick model. Grey surface indicates the fused FAD and substrate binding pocket. Water molecules are shown as red spheres. b) Cross‐section of Lsd18 showing the approximate dimensions of the substrate binding pocket. The indicated length (ca. 35 Å) is that of the trajectory from the pocket entrance to the bottom of the substrate binding pocket. The width (ca. 23 Å) is that of the widest point in the pocket. c) Protein environment of FAD. Interaction distances are given in Å. Chloride ion is shown as a green sphere.

The crystal structure of Lsd18 in complex with FAD was solved at 1.51 Å resolution with final *R*_work_ and *R*_free_ values of 0.14 and 0.18, respectively (Figure 2, Table S1). Two molecules of Lsd18 are present in the asymmetric unit. Each Lsd18 contains one FAD and one chloride ion. Like other members of the class A monooxygenase family, Lsd18 consists of a Rossmann‐like FAD binding domain and a β‐strand‐rich substrate binding domain. The substrate binding domain of Lsd18 is filled with water molecules. The FAD forms an extensive hydrogen bond network with Lsd18 (Figure 2c). The phosphate group of FAD interacts with amino acids in the conserved GGGMAG motif, while the ribosyl group interacts with residues in the central β‐stranded sheet. The adenine base makes a π‐cation interaction with the guanidine group of Arg51, and the isoalloxazine ring forms a π‐π stacking interaction with the imidazole group of His71.

Three‐dimensional structure alignment using the Dali server identified MonCI from *Streptomyces cinnamonensis* (RMSD = 1.2 Å, PDB ID: 8T3P) as the closest structural neighbor (Figure S2), followed by para‐hydroxybenzoate hydroxylase (PHBH) from *Paennibacillus* sp. (RMSD = 3.6 Å, PDB ID: 7ON9) and squalene epoxidase from *Homo sapiens* (RMSD = 3.8 Å, PDB ID: 6C6R). All three proteins are flavin‐dependent monooxygenases, and their amino acid sequence identity varies from 19 to 44%. These flavoenzymes are involved in different biological processes. MonCI transforms a triene polyketide chain to a triepoxide during monensin biosynthesis.^[^ ^30^ ^]^ PHBH transforms 4‐hydroxybenzoate to 3,4‐dihydroxybenzoate during degradation of aromatic compounds.^[^ ^31^ ^]^ Squalene epoxidase converts squalene to 2,3(*S*)‐oxidosqualene during cholesterol biosynthesis.^[^ ^32^ ^]^ Interestingly, Lsd18 has a smaller substrate‐binding pocket than MonCI. The substrate‐binding pocket of Lsd18 is ∼35 Å long and ∼23 Å wide at its widest point (Figure 2b). The combined volume of the substrate and FAD binding pockets in Lsd18 is 889 Å^3^, as calculated by CASTp 3.0.^[^ ^33^ ^]^ In comparison, the substrate‐binding pocket of MonCI is ∼40 Å long and ∼25 Å wide, and the combined volume of the two pockets is 995 Å^3^.^[^ ^30^ ^]^ This pocket size difference is consistent with the fact that Lsd18 acts on a smaller substrate than MonCI. The predicted native substrate of Lsd18 is ∼24 Å long when fully extended while the predicted native substrate of MonCI is ∼30 Å long.

The high structural similarity between Lsd18 and the extensively studied PHBH suggests that these two flavoenzymes share a similar catalytic mechanism. In PHBH, binding of the substrate, *p*‐hydroxybenzoate, to PHBH‐FAD triggers the binding of NADPH.^[^ ^34^, ^35^, ^36^ ^]^ Subsequently, FAD is reduced to FADH^−^, and NADP^+^ is released from the enzyme, which completes the reductive half of the reaction cycle. Next, FADH^−^ reacts with molecular oxygen to form the reactive C4a‐hydroperoxyflavin. Finally, a hydroxyl group is transferred from C4a‐hydroperoxyflavin to *p*‐hydroxybenzoate to form 3,4‐dihydroxybenzoate, thereby completing the oxidative half of the reaction cycle. During the reaction cycle, the isoalloxazine ring of FAD undergoes a large swinging motion.^[^ ^37^ ^]^ It assumes the “in” position when FAD interacts with the substrate and the “out” position when it interacts with NADPH. In our Lsd18‐FAD crystal structure, the isoalloxazine ring is in the “in” position, and therefore our crystal structure likely represents the Lsd18 conformation immediately prior to epoxidation. In this crystal structure, the isoalloxazine ring is blocked from transitioning to the “out” position by a chloride ion, which is part of an extensive hydrogen bond network that includes the main chain amide of Gln66, the hydroxyl of Ser309, and three water molecules (Figure 2c). Therefore, chloride ion is expected to act as an inhibitor of Lsd18. To test this idea, we measured the activity of Lsd18 under varying NaCl concentrations (Figure S3). The highest enzyme activity was observed when the reaction buffer contained no NaCl, and the activity decreased as the NaCl concentration was increased from 0 to 300 mM. Our observation is consistent with a previous report which showed that chloride inhibited the hydroxylation activity of PHBH.^[^ ^38^ ^]^

### X‐Ray Crystal Structure of Lsd18‐FAD‐1

**Figure 3:**
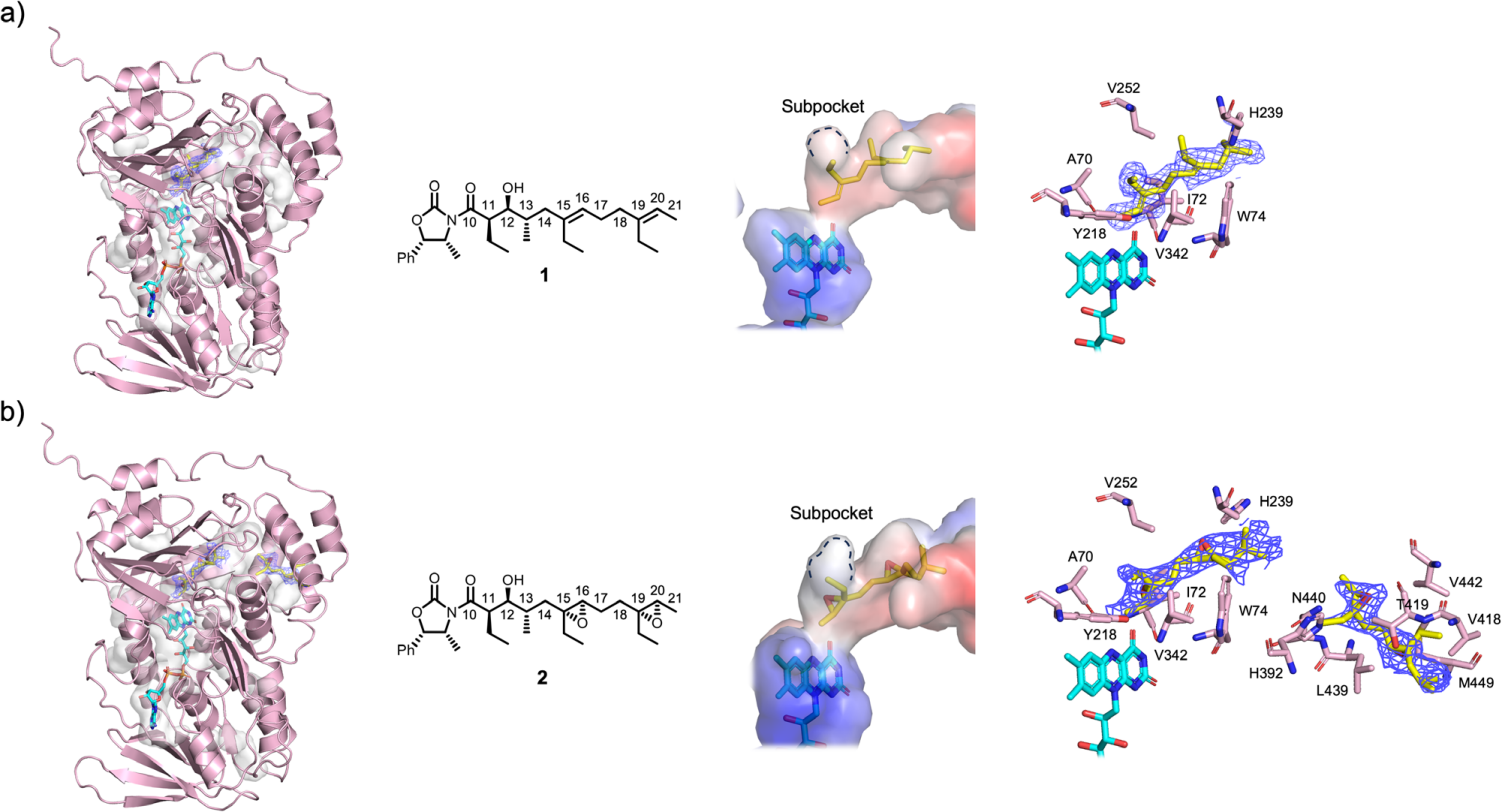
X‐ray crystal structure of Lsd18‐FAD bound to the substrate analogue, **1**, and the product analogue, **2**. a) Structure of Lsd18‐FAD‐**1**. b) Structure of Lsd18‐FAD‐**2**. Colors in the electrostatic potential map indicate positive (blue), neutral (white), and negative (red) surfaces. Electron density for **1** and **2** are shown as a purple mesh (mFo–DFc polder map contoured at 3σ).

To visualize how the substrate molecule binds in the active site, we determined the crystal structure of Lsd18‐FAD in complex with a diene substrate analogue, **1** (Figure 3a). We chemically synthesized **1** following a published method with modifications.^[^ ^20^ ^]^ Lsd18‐FAD‐**1** crystals were prepared by soaking Lsd18‐FAD crystals in a solution containing **1**. The Lsd18‐FAD‐**1** crystal structure was solved at 1.85 Å resolution with final *R*_work_ and *R*_free_ values of 0.16 and 0.20, respectively (Figure 3a, Table S1). Two Lsd18 molecules are present in the asymmetric unit. Each Lsd18 contains one FAD, one substrate analogue, and one chloride ion. No electron density was observed for the oxazolidinone head group of **1**, and therefore it was not modeled. The conformation of Lsd18 in the Lsd18‐FAD‐**1** complex structure is nearly identical to that in the Lsd18‐FAD structure (RMSD = 0.13 Å).

**1** is bound adjacent to the FAD isoalloxazine ring and makes contact with Lsd18 residues A70, I72, W74, Y218, H239, V252, and V342 (Figure 3a). Critically, the *re* face of the C19═C20 alkene of **1** is exposed to the active site cavity. In this configuration, transfer of an oxygen atom from the reactive C4a‐hydroperoxyflavin to the alkene is expected to produce an (*R*,*R*) epoxide, which is consistent with the structure of lasalocid A.

The Lsd18‐FAD‐**1** crystal structure only shows the binding conformation of C19═C20, however we predict that the C15═C16 alkene binds to the active site cavity in a similar manner since it has the same substitution pattern as C19═C20. Both alkenes have the *trans* configuration, and they contain an ethyl substituent at equivalent positions. Therefore, the *re* face of C15═C16 will face the isoalloxazine ring, and consequently an (*R*,*R*) epoxide product will be formed.

### X‐Ray Crystal Structure of Lsd18‐FAD‐2

To visualize how the product molecule interacts with the substrate binding pocket residues, we determined the crystal structure of Lsd18‐FAD bound to a bisepoxide product analogue, **2** (Figure 3b). We chemically synthesized **2** following a published method with modifications.^[^ ^20^ ^]^ Lsd18‐FAD‐**2** crystals were prepared by soaking Lsd18‐FAD crystals in a solution containing **2**. The crystal structure of Lsd18‐FAD‐**2** was solved at 2.20 Å resolution with final *R*_work_ and *R*_free_ values of 0.23 and 0.27, respectively (Figure 3b, Table S1). Two Lsd18 molecules are present in the asymmetric unit. The first Lsd18 molecule contains one FAD, one product analogue molecule, and one chloride ion. However, the second Lsd18 molecule contains one FAD, two product analogue molecules, and one chloride ion. In the latter case, one product molecule is bound at the active site and another product molecule is bound close to the substrate pocket entrance. This 1:2 molar ratio ligand binding is presumably due to the crystal soaking method. No electron density was observed for the oxazolidinone head group of **2**, and it was omitted from the final model. The overall conformation of Lsd18 in the Lsd18‐FAD‐**2** complex is also highly similar to that of the Lsd18‐FAD structure (RMSD = 0.20 Å).

The product analogue, **2**, bound at the Lsd18 active site makes contact with the same set of residues that the substrate analogue, **1**, interacts with in the Lsd18‐FAD‐**1** crystal structure, namely A70, I72, W74, Y218, H239, V252, and V342 (Figure 3b). The second product analogue molecule makes contact with H392, V418, T419, L439, N440, V442, and M449. Binding of two product analogue molecules to Lsd18 is possible because **2** is significantly shorter than the native substrate. During lasalocid A biosynthesis, the pocket space occupied by the two product analogue molecules in the Lsd18‐FAD‐**2** crystal structure is expected to be occupied by a single polyketide substrate.

### Enantioselectivity of Lsd18

The Lsd18‐FAD‐**1** crystal structure provides important clues about the stereocontrol mechanism of Lsd18. In the Lsd18‐FAD‐**1** structure, the isoalloxazine ring of FAD is oriented toward the *re* face of C19═C20. This ensures that the ensuing epoxidation by C4a‐hydroperoxyflavin exclusively produces an epoxide with the (*R*,*R*) configuration. But why does the alkene adopt this orientation in the first place? We propose that the subpocket, formed by Y218, V252, and V342, and the nearby I72, determines the alkene binding orientation (Figure 3a). In our crystal structure, the subpocket accommodates C19‐ethyl of **1**, presumably generating binding free energy and promoting catalysis. The alternative substrate binding conformation, where the *si* face of C19═C20 is oriented toward the isoalloxazine, is not feasible. Our crystal structure shows that this would create a steric clash between the C19‐ethyl and I72 of Lsd18. In conclusion, the subpocket located adjacent to the acitive site dictates that Lsd18 exclusively generates the (*R*,*R*) epoxide product.

**Figure 4:**
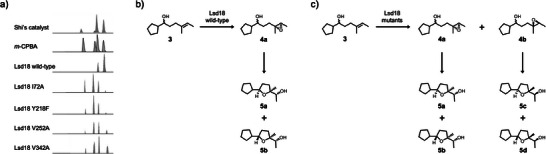
Enantioselectivity of Lsd18. a) Gas chromatography analysis of the cyclic ethers produced by catalytic epoxidation of **3** followed by spontaneous epoxide ring‐opening cyclization. b) The Shi catalyst and wild‐type Lsd18 selectively produce the (*R*,*R*) epoxide product. c) *m*‐Chloroperoxybenzoic acid (*m*‐CPBA), Lsd18 I72A, Y218F, V252A, V342A mutants produce both (*R*,*R*) and (*S*,*S*) epoxide products.

To test our hypothesis, we prepared a series of Lsd18 mutants (I72A, Y218F, V252A, and V342A) and investigated their enantioselectivity using an in vitro assay developed by the Oikawa group (Figure 4, and Figure S4).^[^ ^39^, ^40^ ^]^ In this assay, a monoalkene substrate analogue, **3**, is epoxidated by Lsd18 which then undergoes a non‐enzymatic cyclization to form cyclic ether final products. The cyclic ether products were separated and characterized by gas chromatography‐mass spectrometry (GC‐MS). Since the stereochemistry of the epoxide dictates the stereochemistry of the cyclic ethers that are ultimately formed, GC‐MS analysis of the reaction mixture reveals which epoxide species was initially produced by Lsd18. We found that the wild‐type Lsd18 produced only the (*R*,*R*) epoxide while the I72A, Y218F, V252A, and V342A mutants produced a mixture of (*R*,*R*) and (*S*,*S*) epoxides. This result supports our hypothesis that the wild‐type Lsd18 has a substrate binding pocket that is precisely shaped to promote (*R,R*) epoxide formation. Interestingly, the four Lsd18 mutants also displayed decreased catalytic efficiency compared to wild‐type Lsd18 (Figure S5). This suggests that I72, Y218, V252, and V342 not only control the stereochemical outcome of the epoxidation reaction, but they are also important for maintaining the epoxidation activity of Lsd18.

### Sequential Epoxidation of a Diene by Lsd18

**Figure 5:**
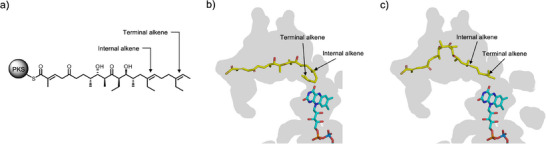
Regiospecificity of Lsd18. a) Presumed native substrate of Lsd18, an undecaketide polyene attached to a polyketide synthase protein. b) Predicted binding mode of the undecaketide during epoxidation of the internal alkene. c) Predicted binding mode of the undecaketide during epoxidation of the terminal alkene.

During lasalocid A biosynthesis Lsd18 transforms both the internal and terminal alkene group in the polyketide substrate into epoxides. Because Lsd18 has just one active site, sequential epoxidation is only possible if the substrate binds at the enzyme active site in at least two different poses. In one pose, the internal alkene must be placed next to the isoalloxazine ring of FAD, and in the other pose the terminal alkene must be placed next to the isoalloxazine ring. We conducted a simple modeling study to determine if the substrate‐binding pocket of Lsd18 is large enough to accommodate both poses. We built a model of the undecaketide substrate (Figure 5a), the product of Lsd16, inside the substrate‐binding pocket of the Lsd18‐FAD crystal structure using the molecular graphics program Coot.^[^ ^41^ ^]^ We fixed the position of the carboxyl end of the undecaketide to the pocket entrance to mimic the immobile nature of the ACP‐tethered undecaketide when bound to Lsd18. Then we adjusted the backbone conformation of the undecaketide so that the terminal alkene is placed in the same location and orientation as the C19═C20 of **1** in the Lsd18‐FAD‐**1** crystal structure. In this model, the undecaketide adopts a “J” conformation (Figure 5b). Next, we repeated the above procedure, but we placed the internal alkene of the undecaketide next to the isoalloxazine ring, again, using the Lsd18‐FAD‐**1** crystal structure as a guide. Here, the undecaketide adopts a “V” conformation (Figure 5c). These models show that the substrate binding pocket of Lsd18 is sufficiently large to accommodate the different conformations required for epoxidation of both the internal and the terminal alkene. Other aspects of the Lsd18 mechanism needs further investigation. It is not known if the undecaketide is released from Lsd18 after the first epoxidation reaction, and the monoepoxide product enters the enzyme again for the second epoxidation reaction. Alternatively, the substrate may get released only after both epoxidations are completed. It is also unclear if the internal alkene or the terminal alkene gets epoxidated first.

## Conclusion

The most significant outcome of this study is the elucidation of the stereocontrol mechanism of Lsd18. The Lsd18‐FAD‐**1** crystal structure has revealed the presence of a subpocket for binding the ethyl substituent of the alkene undergoing epoxidation. This interaction guides the substrate to bind in an orientation that assures (*R*,*R*) epoxide formation and essentially eliminates the possibility of (*S*,*S*) epoxide formation. The Oikawa group has predicted the existence of a stereoselectivity conferring pocket 11 years ago.^[^ ^40^ ^]^ Our work has resolved the location, the size, and the amino acid makeup of this subpocket.

**Figure 6:**
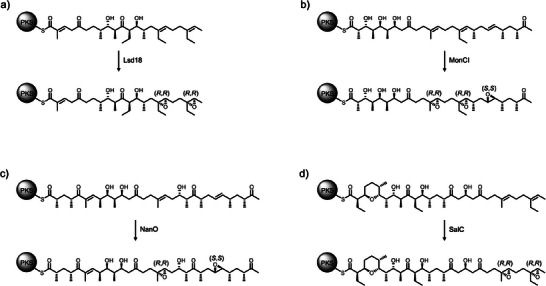
Proposed enzymatic epoxidation reaction in the biosynthesis of a) lasalocid A, b) monensin, c) nanchangmycin, and d) salinomycin.

The stereocontrol mechanism of Lsd18 appears to be a common feature for monooxygenases involved in polyether biosynthesis. Sequence alignment analysis of the monooxygenases from the lasalocid A, monensin, nanchangmycin, and salinomycin biosynthetic pathways shows that the residues that make up the stereoselectivity‐conferring subpocket (Y218, V252, V342) are highly conserved (Figure S6). Interestingly, Lsd18 and SalC exclusively produce (*R*,*R*) epoxides, while MonCI and NanO produce both (*R*,*R*) and (*S*,*S*) epoxides (Figure 6).^[^ ^30^, ^42^, ^43^, ^44^, ^45^ ^]^ This observation can be rationalized by comparing the structure of the respective native polyene substrates. In all cases, a trisubstituted alkene is converted into an (*R*,*R*) epoxide while a disubstituted alkene is transformed into an (*S*,*S*) epoxide. We predict that if the alkene contains a methyl or an ethyl substituent, then it binds at the monooxygenase active site with the *re* face oriented toward the FAD due to the favorable interaction between the substituent group and the subpocket and to avoid a potential steric clash. If the alkene does not have a methyl or ethyl substituent, the *si* face binding is possible and hence an (*S*,*S*) epoxide is formed.

There are still important unanswered questions regarding the mechanism of Lsd18, especially about how it interacts with other proteins in the lasalocid A biosynthetic pathway. For one, the nature of the interaction between Lsd18 and its partner PKS is unknown. This can be addressed by determining the atomic structure of the Lsd18–PKS complex. To do this, we must first determine which of the seven PKSs in the biosynthesis pathway Lsd18 interacts with. Another unknown is how and when the bisepoxide product gets transferred from Lsd18 to Lsd19 for the epoxide hydrolysis reaction. Investigating these aspects of Lsd18 activity is highly challenging due to the difficulty of preparing the native polyene polyketide substrates.

Our findings have the following implications for future Lsd18 engineering studies. First, Lsd18 is expected to have a broad substrate specificity since it does not employ the traditional lock‐and‐key or induced fit ligand binding mechanism. Because Lsd18 has an extra‐large substrate‐binding pocket, it will likely accept a wide variety of polyene substrates. However, Lsd18 may discriminate substrates based on the structure of the ACP domain that the polyene is attached to. Therefore, we must ensure that Lsd18 is able to communicate efficiently with non‐native ACP partners if we are going to generate novel polyethers using engineered biosynthetic pathways. Second, we must enhance the thermostability of Lsd18. We have encountered protein precipitation issues while performing overnight in vitro enzyme activity assays. To address this problem, we have performed structure‐based protein engineering of Lsd18 to improve its stability. We identified an Lsd18 double mutant (T189M/S195 M) that has a 2.7‐fold greater protein expression level, 4.8‐degree higher protein melting temperature, and improved resistance against trypsin digestion, while maintaining the same level of catalytic activity as the wild‐type enzyme.^[^ ^46^ ^]^ Additional rounds of mutagenesis is expected to yield an even more robust form of Lsd18.

Cane, Celmer, and Westley proposed the unified biosynthetic model for polyether biogenesis in 1983.^[^ ^47^ ^]^ This model postulates that all ionophore polyether natural products are generated via a common biochemical strategy that involves construction of an all‐*trans* polyene polyketide, enantioselective epoxidation of the polyketide, and epoxide hydrolysis initiated cyclic ether formation. We have previously reported the structural and biochemical analysis of the Lsd14 polyketide synthase and the Lsd19 epoxide hydrolase.^[^ ^16^, ^17^, ^18^ ^]^ In the current work, we have characterized the structure and function of the Lsd18 monooxygenase. This completes the structural and biochemical characterization of all three central chemical transformations that take place during lasalocid A biosynthesis. The Cane, Celmer, and Westley model was speculative when it was first introduced. The high‐resolution atomic structures of Lsd14, Lsd18, and Lsd19, and the demonstration of their catalytic activity in vitro unequivocally validate the 40‐year‐old model.

## Conflict of Interests

The authors declare no conflict of interest.

## Supporting information



Supporting Information

## Data Availability

The data that support the findings of this study are openly available in Protein Data Bank at https://doi.org/10.2210/pdb8UP4/pdb, https://doi.org/10.2201/pdb8XTZ/pdb, https://doi.org/10.2210/pdb8XU7/pdb reference number 8UP4, 8XTZ, 8XU7.
