# Supplementary material for: Structural Basis of Sequential Enantioselective Epoxidation by a Flavin-Dependent Monooxygenase in Lasalocid A Biosynthesis
Source: Angew Chem Int Ed Engl. Author manuscript; Available in PMC 2026 Jun 10. (PMC12144862; doi:10.1002/anie.202504982)
Supplement: Supinfo [file NIHMS2074820-supplement-Supinfo.docx]

Supporting Information

Structural Basis of Sequential Enantioselective Epoxidation by a Flavin-dependent Monooxygenase in Lasalocid A Biosynthesis

Qian Wang,^†[e]^ Yaming Deng,^†[a]^ Dayan Viera,^†[b]^ Xiaopeng Liu,^[b,d]^ Ning Liu,^[a]^ Yulu Hu,^[a]^ Xiangdong Hu,^[a]^ Hao Wei,^[a]^ Quan Zhou,^[a]^ Ting Lan,^[f]^ Wei He,^[e]^ Xi Chen,*^[a]^ and Chu-Young Kim*^[b,c]^

Corresponding author: chuyoung@illinois.edu; xchen@nwu.edu.cn

Table of Contents

**Materials and Methods3**

Heterologous protein expression and purification3

Protein crystallization, structure determination, and refinement 3

Modeling of hydroperoxyflavin (FAD-OOH)4

Enzyme activity assays4

LC-MS assays4

GC-MS assays5

Chemical synthesis of **1** and **2**5

**Supplementary Table34**

Table S1 Data collection and refinement statistics34

Table S2 Primers for site-directed mutagenesis35

**Supplementary Figures36**

Figure S1 UV-Vis spectrum of Lsd1836

Figure S2 Structural superposition of Lsd18 and MonCI36

Figure S3 Effect of chloride ion on Lsd18 activity37

Figure S4 SDS-PAGE of recombinant wild-type and mutant Lsd18 proteins37

Figure S5 The expected change in the shape and size of the subpocket38

Figure S6 Comparison of monooxygenases involved in polyethers biosynthesis39

**Materials and Methods**

**Heterologous protein expression and purification**

Production of Lsd18 and its mutants from *Streptomyces lasalocidi* was partly based on the procedure described elsewhere (PMID: 22506807). Briefly, pCold-based vector carrying the *lsd18* gene and the chaperone-encoding vector pG-KJE8 (Takara Bio) were introduced into *Escherichia coli* strain BL21(DE3). The culture was grown in Luria Broth medium to OD_600nm_ of 0.6 with 100 $\mu$g/mL ampicillin and 25 $\mu$g/mL chloramphenicol. Lsd18 was induced by 100 $\mu$M isopropyl-$\beta$-D-galactoside, 0.5 mg/mL L-arabinose and 5 ng/mL tetracycline. The culture was incubated for another 20 hours at 15 $℃$. Cells were harvested by centrifugation, resuspended in 50 mM sodium phosphate pH 7.4, 300 mM sodium chloride, 40 mM imidazole, 10% (v/v) glycerol and lysed by sonication. After centrifugation at 18,000 g for 40 min, the cleared supernatant was loaded onto a HisTrap column and washed with a wash buffer containing 50 mM sodium phosphate pH 7.4, 300 mM sodium chloride, 80 mM imidazole, 10% (v/v) glycerol. N-terminal His-tagged Lsd18 were eluted with a wash buffer supplemented with 200 mM imidazole. Fractions containing Lsd18 were diluted by a buffer containing 20 mM Tris pH 8.5, 10% (v/v) glycerol and further purified by anion exchange chromatography using a HiTrapQ column followed by gel filtration on a Superdex75 10/300 GL column (Cytiva) in 20 mM Tris pH 8.5, 100 mM sodium chloride. The protein was >95% pure as determined by polyacrylamide gel electrophoresis, and the yield was approximately 5 mg per liter of culture. The sample was further concentrated to 5.5 mg/mL for long-term storage at -80 celsius.

**Protein crystallization, structure determination, and refinement**

Attempts to crystallize unmodified Lsd18 was unsuccessful. We could only obtain Lsd18 crystals suitable for X-ray analysis after alkylating the surface Lys residue by treating the protein with dimethylamine borane and acetaldehyde. Crystals were obtained from a 1:1 mixture of protein solution (5.5 mg/mL in 20 mM Tris pH 8.5, 100 mM sodium chloride) and a reservoir solution (0.1 M imidazole pH 8, 1 M sodium chloride, 32% (w/v) PEG8000) by the hanging-drop vapor diffusion method at 18 $℃$. The crystals were transferred into a cryoprotectant containing 0.1 M Tris pH 8.5, 0.85 M NaCl, 32% (w/v) PEG4000, and flash-frozen in liquid nitrogen until X-ray data collection on beamline 17-ID at the Advanced Photon Source, Argonne National Laboratory (Argonne, Illinois, USA). All diffraction data were indexed, integrated and scaled with the autoPROC (PMID: 21460447). The initial phases were determined by molecular replacement using MOLREP from CCP4. The crystal structure of MonCI (PDB: 8T3P) was used as a search model. Refinement was performed with Phenix.refine (PMID: 22505256) followed by manual examination and rebuilding of the refined coordinates in the program Coot (PMID: 20383002).

The crystals of Lsd18 in complex with the substrate, or product condition was 0.1 M Tris pH 7.5, 1.0 M NaCl, 32% (w/v) PEG8000, 4% tert-butanol and the crystals of the complex were obtained by soaking. The concentration of Lsd18 was 5.8 mg/mL and the substrate or product was dissolved in DMSO with the final concentration of 25 mM. The soaking buffer contained the crystallization buffer with 10% DMSO and 2.5 mM substrate or product. The crystals were soaked for 30 min and were then transferred into a cryoprotectant containing 80% mother liquor with 20% glycerol, and flash-frozen in liquid nitrogen until X-ray data collection on beamline BL18U1 at the Shanghai Synchrotron Radiation Facility (Shanghai, China). Data were processed and scaled by HKL3000 (PMID: 16855301) and aimLess (PMID: 37259835) and the structures were solved by molecular replacement with the program MOLREP (PMID: 37259835) using Lsd18-apo (PDB: 8UP4) as the template. Refinement was performed with Phenix.refine (PMID: 22505256) and Refmac (PMID: 37259835) followed by manual examination and rebuilding of the refined coordinates in the program Coot (PMID: 20383002).

**Enzyme activity assays**

For substrate **3**, the enzymatic assay was as the following: the reaction mixture consisted of 80 µM substrate, 2 mM NADH, 2 mM NADPH, 80 µM FAD, 15 µM Lsd18, 15 µM Fre, 15 µM Lsd19, 30% DMSO, 300 mM NaCl, 5% glycerol and 50 mM Tris-HCl pH 8.0 buffer. The mixture was incubated at 30 $℃$ for 4 hours. Then ethyl acetate was used to extract the substrate and product and evaporated to obtain the sample for GC-MS analysis.

For substrate **1** and farnesyl acetate, the enzymatic assay was as the following: the reaction mixture consisted of 80 µM substrate, 1 mM NADH, 1 mM NADPH, 80 µM FAD, 15 µM Lsd18, 15 µM Fre, 15 µM Lsd19, 30% methanol, 300 mM NaCl, 5% glycerol and 50 mM Tris-HCl pH 8.0 buffer. The mixture was incubated at 30 $℃$ for 16 hours. Then ethyl acetate was used to extract the substrate and product and evaporated to obtain the sample for LC-MS analysis.

**LC-MS assays**

The LC-MS experiment for substrate **1** was preformed using SHIMADZU LC-30AD as the liquid chromatography and Thermo Scientifical Q EXACTIVE as the Mass spec. The HPLC column was ACQUITY UPLC® HSS T31.8 µM 2.1x100 mm ColµMn. The temperature of the column was set to 40 $℃$ and the flow rate was 0.4 mL/min. The buffer for pump A was 99.9% H_2_O and 0.1% formic acid while the buffer for pump B was 99.5% methanol and 0.05% formic acid. The program was set as: 12% B for 0.01minute, 12% - 20% B from 0.01 to 2 minute, 20% - 80% B from 2 to 3 minutes, 80% - 95% B from 3 to 6 minutes, 95%B from 6 to 9 minutes, 95%-20% B, from 9 to 11 minutes. The mass scanning scope was 150-600 m/z. For the products converted from substrate **1**, 470.3257-492.3075 ^+^ m/z，486.3206-508.3025 ^+^ m/z were chosen for analysis.

The LC-MS experiment for substrate farnesyl acetate was preformed using the instruments with Q Exactive Plus for liquid chromatography and I-Class VION IMS QTof for mass spec. The column used was ACQUITY UPLC® HSS T31.7 µm 2.1x100 mm and the column temperature was set to 35°C. The buffer for pump A was 99.9% H_2_O and 0.1% formic acid while the buffer for pump B was 100% acetonitrile. The program was set as: 10% B at time 0, 10% - 20% B from 0 to 1 minute, 20% - 85% B from 1 to 2 minutes, 85% - 100% B from 2 to 3 minutes, 100% B from 3 to 4 minutes, 100%-5% B, from 4 to 4.5 minutes, 5% B from 4.5 to 6 minutes. The mass scanning scope was 50-2000 m/z. For the products converted from **9**, peak 265.21620-287.1977 ^+^ m/z，281.2111-303.1907 ^+^ m/z and 297.2060-319.3012m/z were chosen for analysis.

**GC-MS assays**

The instrument used for GC-MS is Agilent Technologies 7820A and column was CHIRAMIX capilary (0.25 mm×30 m, 0.25 µm, InterCap). The method is as the following: first, heat at 40 $℃$ for 3 minutes, then increase the temperature from 40 $℃$ to 110 $℃$ with the rate of 3 $℃$/min, next, increase the temperature from 110 $℃$ to 140 $℃$ with the rate of 0.5 $℃$/min, finally keep the temperature at 140 $℃$ for 3 minutes. The flow rate of helium gas was 0.67 mL/min.

**Chemical synthesis of 1 and 2**

All reactions that required anhydrous conditions were carried out by standard procedures under N_2_ atmosphere. Commercially available reagents were used as received. The solvents were dried by distillation over the appropriate drying reagents. Reactions were monitored by TLC. ^1^H NMR and ^13^C NMR spectra were recorded on 400 MHz or 600 MHz Bruker spectrometers. Chemical shifts of ^1^H NMR were reported in part per million relative to the CDCl_3_ residual peak (δ 7.26). Chemical shifts of ^13^C NMR were reported relative to CDCl_3_ (δ 77.16). The abbreviations are used as follows: s (singlet), d (doublet), t (triplet), quart. (quartet), quint. (quintet), m (multiplet), br (broad). High resolution mass spectrometry (HRMS) data were obtained on a Micro TOF-QⅡ (hybrid quadrupolar/time-of flight) API US system by electrospray ionization (ESI) in the positive ion mode or negative ion mode using a Bruker instrument. Reactions were followed with TLC (0.254 mm silica gel 60-F plates). Visualization was accomplished with UV light. Flash chromatography separations were performed on 200-300 mesh silica gel.

S1. Preparation of (4*R*,5*S*)-4-methyl-5-phenyl-3-((2*R*,3*S*,4*S*,6*E*,10*E*)-2,6,10-triethyl-3-hydroxy-4-methyldodeca-6,10-dienoyl)oxazolidin-2-one

*S1.1 Preparation of Tert-butyl(pent-4-yn-1-yloxy)diphenylsilane* (**S1**)

Pent-4-yn-1-ol (1.38 mL), CH_2_Cl_2_ (30 mL) and imidazole (1.320 g) were added to a 250 mL flask at 0°C followed by dropwise addition of TBDPSCl (4.11 mL). The reaction was stirred for 1 h at 0°C and then quenched with CH_2_Cl_2_ followed by extraction with CH_2_Cl_2_. The combined organic layers were washed with NaHCO_3_, then dried over Na_2_SO_4_ and concentrated in *vacuo*. The residue was purified by flash chromatography (silica, 10% ethyl acetate/hexane) to yield **S1** 4.67 g (98%).

^1^H NMR (400 MHz, CDCl3) δ 7.68 (dd, *J* = 7.9 Hz, 4H), 7.49 – 7.32 (m, 6H), 3.76 (t, *J* = 6.0 Hz, 2H), 2.36 (m, *J* = 7.2 Hz, 2H), 1.93 (t, *J* = 2.7 Hz, 1H), 1.87 – 1.67 (m, 2H), 1.07 (s, 9H). ^13^C NMR (101 MHz, CDCl_3_) δ 135.55, 133.81, 129.57, 127.61, 84.19, 68.28, 62.25, 31.42, 26.83, 19.22, 14.96.

*S1.2 Preparation of (E)-tert-butyldiphenyl((4-((trimethylsilyl)methylene)hexyl)oxy)silane* (**S2**)

**S1** (0.210 g), Pd(PPh3)4 (0.077 g), dioxane(1.5 mL) were added to an oven-dried 25 mL round-bottom flask at N2 atmosphere. Me3SiI (0.18 mL) was then added to this solution at 25°C. Next, a solution of Et2Zn (6.4 mL,1 mol/L in THF) was added dropwise via syringe and the solution changed from brown to clear yellow then back to brown. At the same time, an exothermic phenomenon was observed in this process. The reaction mixture was allowed to react at 25°C for 1 h. After the reaction had finished, moist hexane (50 mL) was added to decompose excess Et2Zn. The solvent in mixture was evaporated in *vacuo* and became brown oil, which was purified by chromatography (silica, 100% hexane) to afford **S2** 0.124 g (47%) as colorless oil.

^1^H NMR (400 MHz, CDCl3) δ 7.70 (dd, *J* = 7.7Hz, 4H), 7.42 (m, *J* = 6.7 Hz, 6H), 5.18 (s, 1H), 3.69 (t, *J* = 6.4 Hz, 2H), 2.16 (m, *J* = 7.4 Hz, 4H), 1.77 – 1.64 (m, 2H), 1.08 (s, 9H), 1.03 (d, *J* = 7.5 Hz, 3H), 0.11 (s, 9H). ^13^C NMR (101 MHz, CDCl_3_) δ 151.53, 135.94, 134.48, 129.86, 127.94, 107.96, 64.02, 32.75, 31.24, 29.17, 27.24, 19.59, 12.71.

*S1.3 Preparation of (E)-tert-butyl((4-(iodomethylene)hexyl)oxy)diphenylsilane* (**S3**)

**S2** (0.212 g) and dry CH3CN(2.5 mL) were added to an oven-dried 25mL round-bottom flask and cooled to 0°C. NIS (0.112 g) was then added in one portion. After stirring at 0°C for 20 min then sodium bicarbonate (50 mL) aqueous solution was added to remove residual NIS. The combined organic solution was dried with Na2SO4 and evaporated to remove the solvent. The resulted product was purified by column chromatography (silica, 100% hexane) to afford **S3** 0.196 g (82%) as faint yellow oil.

^1^H NMR (400 MHz, CDCl3) δ 7.70 (dd, *J* = 7.7 Hz, 4H), 7.42 (m, *J* = 13.7 Hz, 6H), 5.18 (s, 1H), 3.69 (t, *J* = 6.4 Hz, 2H), 2.16 (m, *J* = 7.4 Hz, 2H), 1.77 – 1.64 (m, 2H), 1.08 (s, 9H), 1.03 (d, *J* = 7.5 Hz, 3H). ^13^C NMR (101 MHz, CDCl3) δ 152.81, 135.89, 134.20, 129.95, 128.00, 74.27, 63.32, 33.25, 31.08, 30.84, 27.22, 19.56, 12.02.

*S1.4 Preparation of (E)-tert-butyl((4-ethylhex-4-en-1-yl)oxy)diphenylsilane* (**S4**)


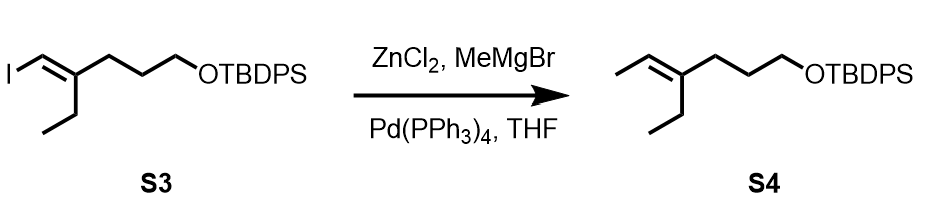


ZnCl2 (2.6 mL, 0.7 mol/L in THF) was treated in an oven-dried 25 mL round-bottom flask at 0°C under N2 atmosphere and CH3BrMg (1.39 mL, 1 mol/L in THF) was added dropwise. Under this condition, the reaction was continued for 3.5 h to generate 1.39 mmol organic Zinc solution. Separately, **S3** (0.200 g), Pd (PPh3)4 (0.025 g) and 4.9 mL dry THF was combined in another round-bottom flask under N2 condition. Decanted organic Zinc (1.39 mmol) solution was added to the iodine/Pb mixture dropwise by syringe at rt. After the consumption of **S3** by TCL (12 h), the reaction was quenched by the addition of H2O and then the layer was separated. The aqueous layer was extracted by ethyl acetate, the combined organic layer was washed by H2O, brine, dried by Na2SO4, filtered and concentrated. After purified by column chromatography (silica, 100% hexane) **S4** 0.123g (80 %) was afforded as colorless oil.

^1^H NMR (400 MHz, CDCl3) δ 7.69 (dd, *J* = 7.8Hz, 4H), 7.50 – 7.34 (m, 6H), 5.16 (q, *J* = 6.7 Hz, 1H), 3.73 – 3.60 (m, 2H), 2.12 – 1.96 (m, 4H), 1.70 – 1.62 (m, 2H), 1.57 (d, *J* = 6.7 Hz, 3H), 1.07 (d, *J* = 3.2 Hz, 9H), 0.95 (t, *J* = 7.6 Hz, 3H). ^13^C NMR (101 MHz, CDCl_3_) δ 141.40, 135.59, 134.17, 129.47, 127.56, 117.83, 63.71, 32.70, 31.21, 26.88, 22.72, 19.23, 12.95, 12.77.

*S1.5 Preparation of (E)-4-ethylhex-4-en-1-ol* (**S5**)

**S4** (0.131g) and 4 mL dried THF were added to a 25 mL round-bottom flask and cooled to 0°C. Tetrabutyl ammonium fluoride (4.2 mL, 1M in THF) was then added dropwise. After being stirred at 0°C for 15 min, the ice bath was removed and the reaction was continued for 3 h at rt before quenched by the addition of H2O. Then the layer was separated and the aqueous layer was extracted by ethyl acetate while the combined organic layer was washed by saturated Sodium bicarbonate solution, brine, dried by Na2SO4, filtered and concentrated in *vacuo*. After purified by column chromatography (silica, 20% ethyl acetate/hexane) **S5** 0.043 g (95%) was afforded as colorless oil.

^1^H NMR (400 MHz, CDCl_3_) δ 5.20 (q, *J* = 6.7 Hz, 1H)，3.62 (t, *J* = 6.5 Hz, 2H), 2.07 (q, 4H), 1.69– 1.61 (m, 2H), 1.57 (d, *J* = 6.7 Hz, 4H), 0.95 (t, *J* = 7.6 Hz, 3H). ^13^C NMR (101 MHz, CDCl_3_) δ141.51, 118.35, 62.90, 33.03, 31.25, 22.86, 13.12, 12.93.

*S1.6 Preparation of (E)-4-ethylhex-4-enal* (**S6**)

1.2 mL oxalyl chloride (2 M in CH2Cl2) and 15 mL dry CH2Cl2 were mixed in a 50 mL over-dried round-bottom flask at -78°C. Then DMSO (0.26 mL) in 2 mL CH2Cl2 was added dropwise via a syringe under an atmosphere of N2. The solution was stirred at -78°C for 20 min and a solution of **S5** (0.151 g) in 3 mL CH2Cl2 was added dropwise via a syringe. After an additional 20 min of stirring at -78°C, the reaction mixture was quenched by adding Et3N (0.5 mL) and was stirred at -78°C for another 15 min before the ice bath was removed. After being stirred for 30 min at rt, the reaction was quenched by adding saturated NaHCO3 solution, and the resulting mixture was extracted with CH2Cl2, the combined organic extracts was washed with water, saturated NaHCO3 solution brine, dried by Na2SO4 and concentrated in *vacuo*. The residue was purified by column chromatography (silica, 20% ethyl acetate/hexane) to afford aldehyde **S6** 0.113 g (76%).

^1^H NMR (400 MHz, CDCl_3_) δ 9.74 (t, *J* = 1.9 Hz, 1H), 5.17 (q, *J* = 6.7 Hz, 1H), 2.52 – 2.47 (m, 2H), 2.35 – 2.29 (m, 2H), 2.07 – 2.00 (m, 2H), 1.57 (d, *J* = 6.7 Hz, 3H), 0.96 (t, *J* = 7.6 Hz, 3H). ^13^C NMR (101 MHz, CDCl_3_) δ 202.88, 139.87, 119.06, 42.45, 28.99, 23.22, 13.13, 12.84.

*S1.7 Preparation of (E)-1,1-dibromo-5-ethylhepta-1,5-diene* (**S7**)

Carbon tetrabromide (1.052 g) and 3.2 mL dry CH2Cl2 were treated in a 25 mL over-dried round-bottom flask and cooled to 0°C. A solution of triphenylphosphine (1.665 g) in 4.4 mL CH2Cl2 was then added dropwise via a syringe. After the addition, the mixture was cooled to -78°C and a solution of **S6** (0.200 g) in 1.6 mL CH2Cl2 was added dropwise. The reaction was stirred at -78°C for 30 min, then at 0°C for 20 min. After the reaction was finished, the mixture was diluted with ethyl acetate and poured into brine. After layers were separated, the aqueous layer was extracted with ethyl acetate, and the combined organic layers were dried over Na2SO4 and concentrated in *vacuo*. The residue was purified by column chromatography (silica, 5% ethyl acetate/hexane) to afford vinyl dibromide **S7** 0.237 g (53%).

^1^H NMR (400 MHz, CDCl_3_) δ 6.34 (t, *J* = 6.9 Hz, 1H), 5.16 (q, *J* = 6.7 Hz, 1H), 2.22 – 2.11 (m, 2H), 2.09 – 1.97 (m, 4H), 1.57 (d, *J* = 6.9 Hz, 3H), 0.96 – 0.91 (m,3H). ^13^C NMR (101 MHz, CDCl_3_) δ 140.28, 138.73, 119.39, 88.77, 34.61, 31.99, 22.89, 13.30, 13.04.

*S1.8 Preparation of (E)-(5-ethylhept-5-en-1-yn-1-yl)trimethylsilane* (**S8**)

Vinyl dibromide **S7** (0.200 g) and THF (7.1 mL) were added to a 25 mL over-dried round-bottom flask and cooled to -78˚C. *n*-BuLi (0.88 mL, 1.6 M in hexanes) was then added dropwise to this mixture via a syringe. After the addition the reaction was reacted for 5 min at -78˚C and 1 h at -25˚C. The reaction was then cooled down again to -78˚C and a solution of TMSCl (0.09 mL) in THF (1.5 mL) was added dropwise. The reaction was then allowed to slowly warm to rt and react for 1 h. After the reaction was finished, the mixture was poured into saturated NH4Cl solution and diluted with ethyl acetate. After layers were separated, the aqueous layer was extracted with ethyl acetate. The combined organic layers were dried over Na2SO4, filtered, and concentrated in *vacuo*. Purification by flash column chromatography (silica, 100% hexane) afforded **S8** 0.127 g (90%).

^1^H NMR (400 MHz, CDCl_3_) δ 5.20 (q, *J* = 6.6 Hz, 1H), 2.30 – 2.26 (m, 2H), 2.23 – 2.18 (m, 2H), 2.04 (q, *J* = 7.7 Hz, 2H), 1.58 (d, *J* = 6.7 Hz, 3H), 0.95 (t, *J* = 7.5 Hz, 3H), 0.14 (s, 9H). ^13^C NMR (101 MHz, CDCl_3_) δ 140.05, 119.03, 107.47, 67.87, 35.65, 25.55, 22.52, 19.33, 12.72.

*S1.9 Preparation of ((1E,5E)-5-ethyl-1-iodohepta-1,5-dien-1-yl)trimethylsilane* (**S9**)


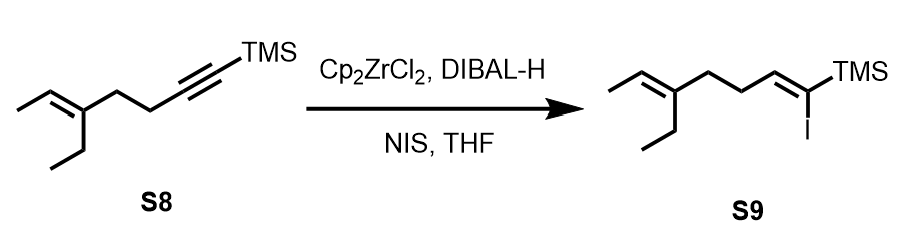


Cp2ZrCl2 (0.262 g) and 3.2 mL THF were added to a 25 mL over-dried round-bottom flask at 0°C. A solution of DIBAL in THF (1 M, 0.89 mL) was then added dropwise by a syringe. The mixture was reacted for 45 min at 0 °C and a solution of **S8** (0.093 g) in 20 mL THF was added dropwise to this suspension. The mixture was warmed to rt and reacted for 6 h to yield a clear yellow solution and then cooled to 0 °C, followed by addition of NIS (0.129 g) in THF (1 mL). After 1 h stirring at 10°C, the solution was quenched by the addition of H2O. The layers were separated and the aqueous layer was extracted with ethyl acetate. The combined organic layers were washed with saturated Na2S2O3, saturated NaHCO3 and brine, dried over MgSO4, and concentrated in *vacuo*. Purification by flash column chromatography (silica, 100% hexane) afforded **S9** 0.215 g (65%) as a colorless oil.

^1^H NMR (400 MHz, CDCl_3_) δ 7.15 (t, *J* = 7.5 Hz, 1H), 5.17 (q, *J* = 13.4, 6.7 Hz, 1H), 2.14–2.16 (q, *J* = 12.8 Hz, 2H), 2.03 (dt, *J* =7.6 Hz, 4H), 1.59 (d, *J* = 6.4 Hz, 3H), 1.02 – 0.90 (m, 3H), 0.27 (s, 9H). ^13^C NMR (101 MHz, CDCl_3_) δ 156.41, 140.56, 119.08, 106.70, 36.10, 34.33, 23.08, 13.30, 13.07, 1.43.

*S1.10 Preparation of ((3Z,7E)-7-ethylnona-3,7-dien-3-yl)trimethylsilane* (**S10**)

ZnCl_2(_2.2 mL, 0.7 mol/L in THF) was treated in an oven-dried 25 mL round-bottom flask at 0°C under N2 atmosphere and EtMgBr ( 1.6 mL, 1 mol/L in THF) was added dropwise by a syringe. The mixture was then reacted for 0.5 h at 0°C, 3 h at rt to generate organic Zinc solution. Separately, **S9** (0.10 g), Pd (PPh3)4 (18 mg) and dry 1 mL THF were combined in another round-bottom flask under N2 condition. Decanted solution of organic Zinc was added to the **S9**/Pd mixture made above by a syringe dropwise at rt. After the consumption of **S9** by TCL (12 h), the reaction was quenched by adding H2O and then the layer was separated. The aqueous layer was extracted by ethyl acetate while the combined organic layer was washed by H2O, brine, dried by Na2SO4, filtered and concentrated in *vacuo*. After purified by column chromatography (silica, 100% hexane) **S10** 0.669 g (96%) was afforded as colorless oil.

^1^H NMR (400 MHz, CDCl_3_) δ 5.95 (t, *J* = 6.9 Hz, 1H), 5.20 (q, *J* = 13.0 Hz, 1H), 2.21 (q, *J* = 7.5 Hz, 2H), 2.12 – 2.00 (m, 6H), 1.61 (t, *J* = 6.5 Hz, 3H), 0.95-1.00 (m, *J* = 12.1 Hz, 6H), 0.16 (s, *9*H). ^13^C NMR (101 MHz, CDCl_3_) δ 141.69, 141.16, 118.25, 37.37, 31.29, 30.06, 23.21, 15.79, 13.27, 13.15, 0.61.

*S1.11 Preparation of (2E,6E)-3-ethyl-7-iodonona-2,6-diene* (**S11**)

The reaction flask with the solution of **S11**(0.06 g) in 1.32 mL dry DMF at 0°C was protected from light. A solution of NIS (0.072 g) in 1 mL DMF was added dropwise by a syringe. After reacted at 0°C for 20 min and then rt for 3 h, the mixture was quenched by the addition of saturated Na2S2O3 solution after which the layer was separated. The aqueous layer was extracted by CH2Cl2 while the combined organic layer was washed by H2O, brine, dried by Na2SO4, filtered and concentrated in *vacuo*. After purified by column chromatography (silica, 100% hexane) **S11** 0.049 g (55%) was afforded as colorless oil.

^1^H NMR (400 MHz, CDCl_3_) δ 6.12 (t, *J* = 7.3 Hz,1H), 5.18 (q, *J* = 6.8 Hz, 1H), 2.39 (q, *J* = 7.4 Hz, 2H), 2.15 – 2.08 (m, 2H), 2.07 – 1.97 (m, 4H), 1.59 (d, *J* = 6.8 Hz, 3H), 1.04 (t, *J* = 6.5 Hz, 3H), 0.96 (d, *J* = 8.2, 7.0 Hz, 3H). ^13^C NMR (101 MHz, CDCl_3_) δ 140.77, 140.41, 118.96, 105.41, 36.07, 32.60, 30.00, 23.02, 14.66, 13.28, 13.06.

*S1.12 Preparation of (4R,5S)-3-((2R,3R)-2-ethyl-3-hydroxy-4-methylpent-4-enoyl)-4-methyl-5-phenyloxazolidin-2-one* (**P1**)

*n*-Bu2BOTf (0.182 g) was slowly added to a stirred solution of (4*R*,5*S*)-3-butyryl-4-methyl-5-phenyloxazolidin-2-one (0.15 g) in 2 mL dry CH2Cl2 at 0°C under N2 dropwise. After reacted for 15min at 0°C, (*i*-Pr)2NEt (0.1 mL) was added dropwise over 20 min. The mixture was continued to react for a further 2 h then the reaction mixture was cooled to -78°C and a solution of methacrolein (0.043 g) in 2 mL CH2Cl2 was added dropwise. The reaction was continued to be stirred at -78°C for another 2 h, rt for 12 h. The reaction mixture was then quenched by the addition of pH 7 phosphate buffer (1 mL) in 2 mL MeOH. After the mixture was reacted for 10 min at 0°C, a solution of 30% aqueous hydrogen peroxide (0.4 mL) in MeOH (0.8 mL) was added slowly. After reacted for 1 h at 0°C, excess peroxides were quenched with aqueous FeSO4 and the resulting solution was concentrated in *vacuo* to remove excess MeOH. The aqueous residue was extracted with ethyl acetate and the combined organic extracts were washed successively with 5% aqueous NaHCO3 and brine before being dried (Na2SO4), filtered and concentrated in *vacuo*. After purified by column chromatography (silica, 20% ethyl acetate/hexane) **P1** 0.160 g (83%) was afforded as colorless oil.

^1^H NMR (400 MHz, CDCl_3_) δ 7.44 – 7.36 (m, 3H), 7.31 (d, *J* = 1.3 Hz, 2H), 5.65 (d, *J* = 7.3 Hz, 1H), 5.09 (s, 1H), 4.95 (s, *J* = 1.0 Hz, 1H), 4.83-4.76(m, 1H), 4.35 (d, *J* = 4.5 Hz, 1H), 4.22 – 4.07 (m, 1H), 1.82-1.78 (m, 6H), 0.92-0.89(m, 6H). ^13^C NMR (101 MHz, CDCl_3_) δ 175.99, 153.11, 144.74, 133.41, 129.09, 129.00, 112.29, 79.07, 75.27, 55.30, 47.26, 19.94, 19.23, 14.75, 11.87.

*S1.13 Preparation of (4R,5S)-3-((2R,3R)-2-ethyl-4-methyl-3-((triethylsilyl)oxy)pent-4-enoyl)-4-methyl-5-phenyloxazolidin-2-one* (**P2**)

**P1** (0.150 g) and 3 mL dry CH2Cl2 were treated in an oven-dried 25 mL round-bottom flask at 0°C. Then imidazole (0.048 g) was added and the mixture was stirred for 5 min at 0°C. Triethylchlorosilane (118 μl) was added dropwise by a syringe and the reaction was continued for 2 h at the same temperature. After the reaction was completed, the solvent was evaporated in *vacuo* and the product purified by flash column chromatography (silica, 20% ethyl acetate/hexane) to afford **P2** 0.200 g (98%) as colorless oil.

^1^H NMR (400 MHz, CDCl_3_) δ 7.45 – 7.36 (m, 3H), 7.30 (d, *J* = 7.7 Hz, 2H), 5.55 (d, *J* = 7.1 Hz, 1H), 4.84 (d, *J* = 13.0 Hz, 2H), 4.68 (q, *J* = 6.6 Hz, 1H), 4.31 (d, *J* = 8.3 Hz, 1H), 1.98 – 1.77 (m, 1H), 1.75 (s, 5H), 1.01 – 0.84 (m, 15H), 0.60 (q, *J* = 7.8 Hz, 6H). ^13^C NMR (101 MHz, CDCl_3_) δ 174.36, 152.62, 146.33133.15, 128.56, 125.46, 112.35, 78.59, 78.00, 55.11, 49.31, 22.20, 17.03, 14.30, 11.42, 6.76, 4.63.

*S1.14 Preparation of (4R,5S)-4-methyl-5-phenyl-3-((2R,3S,4S,6E,10E)-2,6,10-triethyl-4-methyl-3-((trimethylsilyl)oxy)dodeca-6,10-dienoyl)oxazolidin-2-one* (**P3**)

**P2** (0.150 g) was treated in an oven-dried 25 mL round-bottom flask and 9-BBN diner in 2.2 mL THF was added dropwise by a syringe at 0°C under N2 atmosphere. After the addition, the mixture was stirred for 6 h at the same temperature and then 3 M Cs2CO3 solution (0.24 mL) was added to this mixture and raised the temperature to rt. The reaction was continued for 1 h at rt and then vinyl iodide **S11** (0.044 g) in a mixed solvent 3.5 mL (THF: dioxane : H_2_O=10:8:1,v/v) and Pd(dppf)Cl2 (0.032 g) were added. The mixture was stirred for 16 h at rt. After the reaction was completed, the mixture was quenched by adding H2O and the layer was separated. The aqueous layer was extracted by ethyl acetate. The combined organic layer was washed with brine, dried over Na2SO4 and concentrated in *vacuo*. The residue was purified by flash column chromatography (silica, 15% ethyl acetate/hexane) to afford **P3** 0.138 g (68%).

^1^H NMR (400 MHz, CDCl_3_) δ 7.44 – 7.35 (m, 3H), 7.34 – 7.29 (m,2H), 5.56 (d, *J* = 7.0 Hz, 1H), 5.21 – 5.13 (m, 1H), 5.04 (t, *J* = 6.8 Hz, 1H), 4.80 – 4.75 (m, 1H), 3.93 (dd, *J* = 7.5, 2.8 Hz, 1H), 2.36 – 2.25 (m, 1H), 2.11-2.00 (m, 8H), 1.88 – 1.69 (m, 5H), 1.57 (t, *J* = 6.8 Hz, 3H), 1.02-0.95 (m, 24H), 0.66-0.64(m, 6H). ^13^C NMR (101 MHz, CDCl_3_) δ 175.81, 152.74, 141.76, 139.45, 133.47, 128.96, 126.05, 125.83, 118.15, 78.91, 55.58, 48.04, 38.63, 36.66, 26.76, 23.33, 23.07, 20.22, 16.55, 14.59, 13.20, 13.06, 11.57, 7.35, 5.76.

*S1.15 Preparation of (4R,5S)-4-methyl-5-phenyl-3-((2R,3S,4S,6E,10E)-2,6,10-triethyl-3-hydroxy-4-methyldodeca-6,10-dienoyl)oxazolidin-2-one* (**1**)

**P3** (0.010 g), MeCN (0.8 mL), pyridine (0.8 mL) were added to an oven-dried 25 mL round-bottom flask and cooled to 0°C. A solution of HF·Py (0.152 mL) was then slowly added to the mixture, which was stirred for 6 h at rt and then quenched with ice water. After the layer was separated, the aqueous layer was extracted with CH2Cl2 while the organic layer was washed with NaHSO4 (2 M) to pH2.0, then with aqueous NaHCO3, and finally washed with brine (40 mL), dried over Na2SO4, and concentrated in vacuo, the crude reaction mixture was purified by flash column chromatography (silica, 30% ethyl acetate/hexane) and further purified by reverse phase HPLC (UniSil^®^10-120 C18 Ultra 10 um 21.2x250 mm UV at 215 nm. Washing conditions: a gradient of 20-95% MeOH for 30 min, then 95 MeOH for 30 min, 15 mL/min) to give the target product 0.064 g (79%).

^1^H NMR (400 MHz, CDCl_3_) δ 7.45 – 7.37 (m, 3H), 7.31 (d, *J* = 6.9 Hz, 2H), 5.65 (d, *J* = 7.0 Hz, 1H), 5.25 – 5.02 (m, 2H), 4.86 – 4.77 (m, 1H), 4.21 – 4.11 (m, 1H), 3.66 – 3.54 (m, 1H), 2.58 (d, *J* = 13.2 Hz, 1H), 2.16 – 1.95 (m, 8H), 1.94 – 1.85 (m, 2H), 1.82 – 1.68 (m, 3H), 1.57 (d, *J* = 6.5 Hz, 3H), 0.95 (m, 12H), 0.89 – 0.84 (m, 3H). ^13^C NMR (101 MHz, CDCl_3_) δ 176.80, 152.96, 141.78, 139.82, 133.42, 129.06, 126.58, 125.89, 118.21, 79.01, 77.50, 55.34, 46.74, 37.39, 37.21, 26.75, 23.23, 23.01, 20.01, 15.92, 14.75, 13.52, 13.25, 11.98.

S2. Preparation of (4*R*,5*S*)-3-((2*R*,3*S*,4*S*)-2-ethyl-5-((2*R*,3*R*)-2-ethyl-3-(2-((2*R*,3*R*)-2-ethyl-3-methyloxiran-2-yl)ethyl)oxiran-2-yl)-3-hydroxy-4-methylpentanoyl)-4-methyl-5-phenyloxazolidin-2-one

*S2.1 Preparation of* *(4R,5S)-3-((2R,3S,4S)-2-ethyl-5-((2R,3R)-2-ethyl-3-(2-((2R,3R)-2-ethyl-3-methyloxiran-2-yl)ethyl)oxiran-2-yl)-4-methyl-3-((trimethylsilyl)oxy)pentanoyl)-4-methyl-5-phenyloxazolidin-2-one* (**P4**)

Shi’s catalyst (D-fructose) (46 mg), buffer [0.05 M solution of Na2B4O7**^.^**10H_2_O in 4×10^-4^ M aqueous Na_2_(EDTA), 2.9 mL] and Bu4NHSO4 (4.1 mg, 12.1 µmol) were added to a stirred solution of **P3** (96 mg) in CH3CN-dimethoxymethane (10.2 mL, 2:1, v/v). After the mixture was cooled to 0˚C, a solution of Oxone (255 mg) in aqueous Na2(EDTA) (4×10^-4^ M, 4.8 mL) and a solution of 0.9 M K2CO3 (4.8 mL) were added separately with a syringe pump over a period of 2 h. At this point, the mixture was diluted with water and extracted with ethyl acetate. The combined organic layers were washed with brine, dried over anhydrous Na2SO4, and concentrated in *vacuo*. Purification of the residue by flash column chromatography (silica, hexane/ethyl acetate 20/1 to 4/1) to afford **P4**.

*S2.2 Preparation of (4R,5S)-3-((2R,3S,4S)-2-ethyl-5-((2R,3R)-2-ethyl-3-(2-((2R,3R)-2-ethyl-3-methyloxiran-2-yl)ethyl)oxiran-2-yl)-3-hydroxy-4-methylpentanoyl)-4-methyl-5-phenyloxazolidin-2-one* (**2**)

1 M n-Bu4NF in THF (4.9 µL) was added to a stirred solution of **P4** (5.11 mg) in 1.5 mL dry THF. After the resulting solution was stirred at 25˚C for 30 min, the mixture was poured into saturated aqueous NaHCO3 and then extracted with ethyl acetate. The combined organic layers were washed with brine, dried over anhydrous Na2SO4, and concentrated in *vacuo*. Purification of the residue by flash column chromatography (silica , hexane/ ethyl acetate = 10/1 to 2/1 containing 1% Et_3_N) afford the **2**.

HRMS (ESI) m/z: [M+Na]^+^ calculated for C_29_H_43_NO_6_^+^ 524.3090, found 524.2999

*S2.3 Preparation of* **P5** (epoxide **4a+4b**)

(*E*)-1-cyclopentyl-4-methylhex-4-en-1-ol (0.320 g) and 12 mL of CH_2_Cl_2_ was were mixed in a 100 mL over-dried round-bottom flask, then 80% of *m*-CPBA (0.540 g) in CH_2_Cl_2_ was added at 0°C under a nitrogen atmosphere, and the mixture was then stirred at rt for 1 h. after the reaction was finished, A 1 M aqueous solution of sodium hydroxide (5 mL) was added to the solution, and the organic layer was separated. The aqueous layer was extracted with dichloromethane. The combined organic layer was washed with brine, dried over anhydrous magnesium sulfate, and concentrated in vacuo. The residue was purified by column chromatography (silica, hexane/ethyl acetate = 15:1) to afford a mixture product (**5a**, **5b**, **5c**, **5d**) (0.180 g, 51% yield) as colorless oil, which is generated from nonenzymatic cyclization of epoxide **4a+4b**.

*S2.4 Preparation of* **P6** (epoxide **4a**)

(*E*)-1-cyclopentyl-4-methylhex-4-en-1-ol (0.200 g) was dissolved in MeCN (5.5 mL) and DMM (11 mL), Shi’s D-ketone (0.171 g), *n*-Bu_4_NHSO_4_ (0.030 g), and buffer (11 mL, 5.00 × 10^-2^ M solution of Na_2_B_4_O_7_·10H_2_O in 4.00 × 10-4 M aqueous Na_2_(EDTA) were added to the solution. A solution of Oxone (1.87 g) in aqueous Na_2_(EDTA) (5.5 mL, 4.00 × 10^-4^ M) and a solution of K_2_CO_3_ (1.77 g) in 5.5 mL H_2_O were added dropwise separately to the solution at 0°C over a period of 1 h via additional funnels, and the mixture was stirred for 30 min. The mixture was diluted with H_2_O and extracted with CH_2_Cl_2_. The organic layers were washed with brine, dried over anhydrous Na_2_SO_4_, filtered, and concentrated in vacuo. The residue was purified by flash column chromatography (silica , hexane/ethyl acetate = 15:1) to afford **P6** (**5a**, **5b**) 0.179 g (82%) as colorless oil, which is generated from nonenzymatic cyclization of epoxide **4a**.

**S1** NMR spectra

^1^H NMR (400 MHz, CDCl_3_)


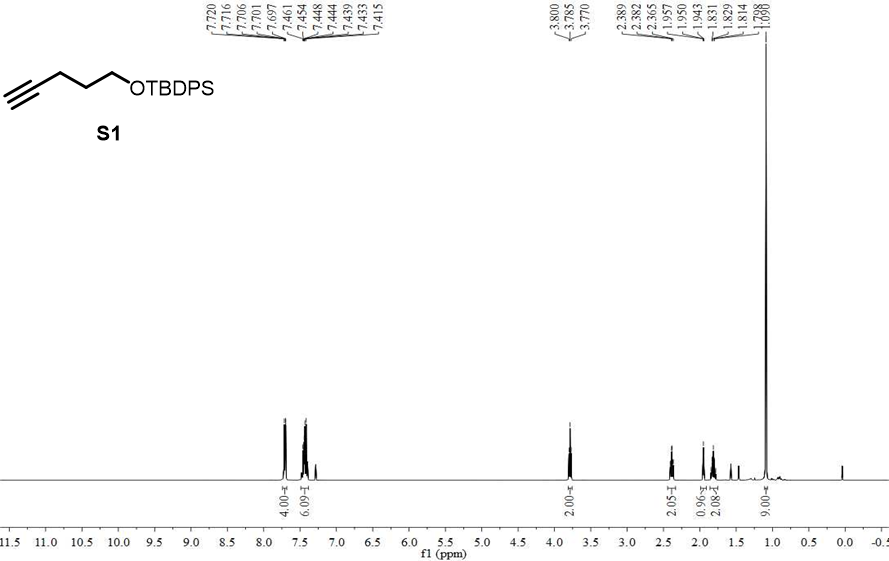


^13^C NMR (101 MHz, CDCl_3_)


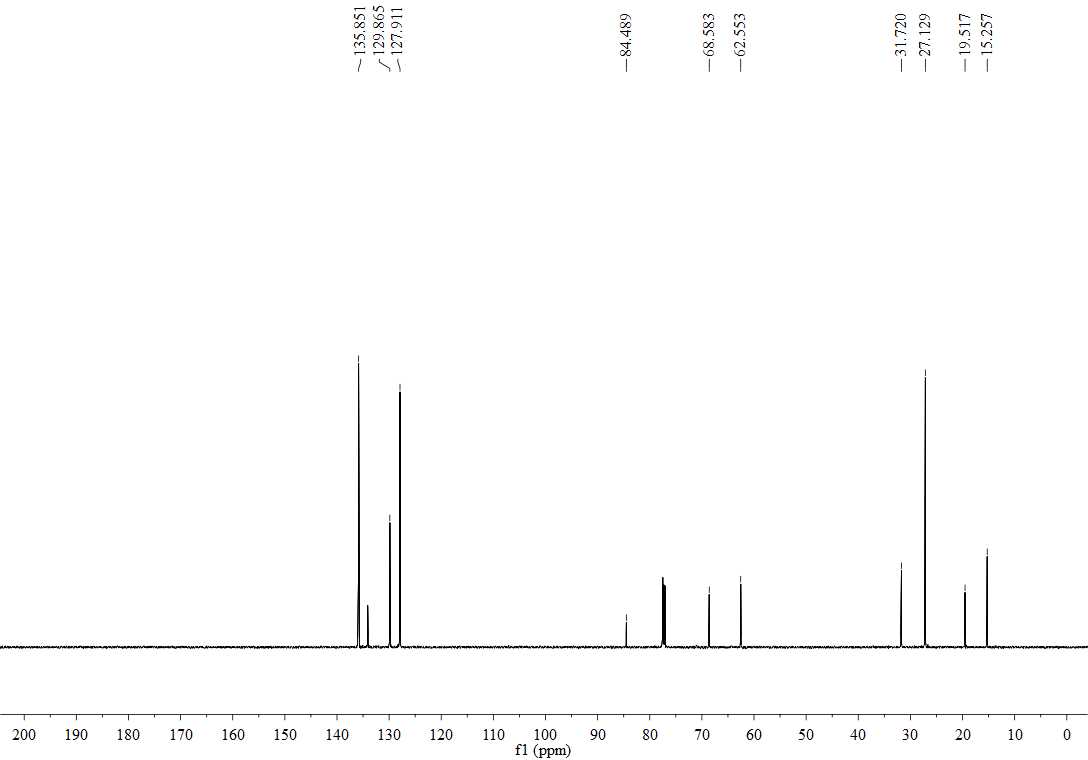


**S2** NMR spectra

^1^H NMR (400 MHz, CDCl_3_)


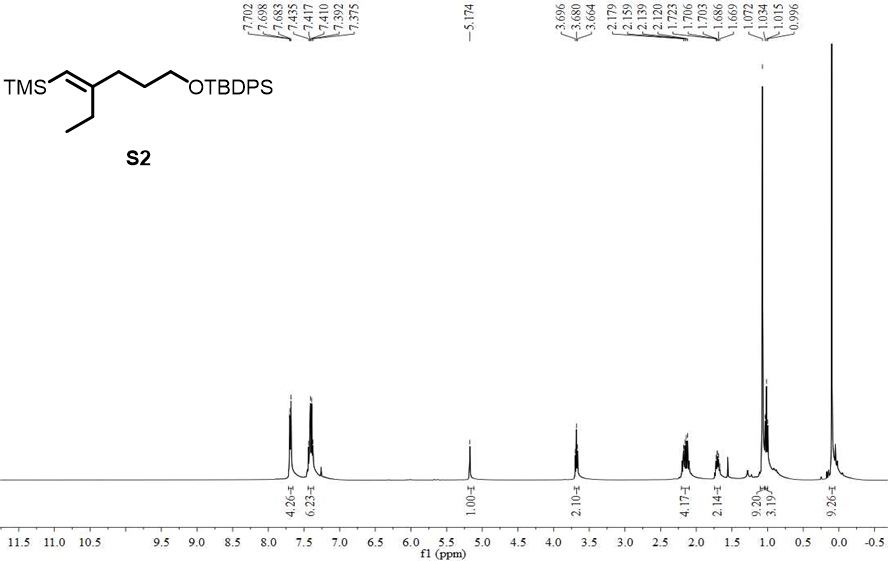


^13^C NMR (101 MHz, CDCl_3_)


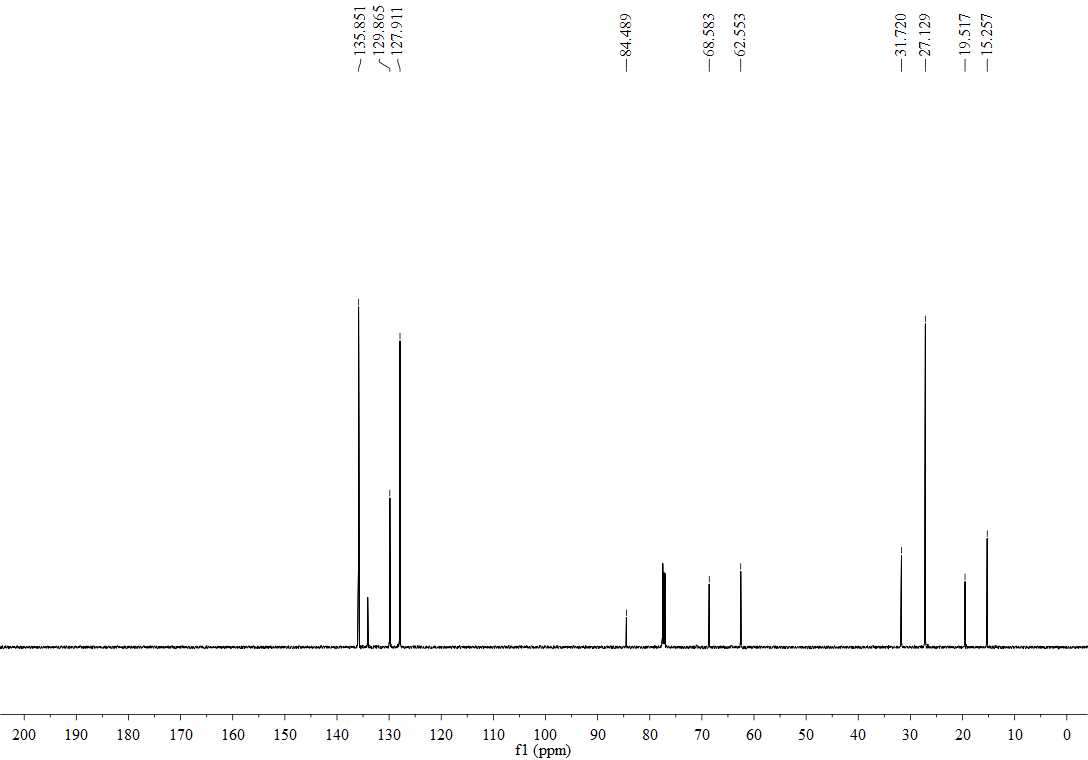


**S3 NMR spectra**

^1^H NMR (400 MHz, CDCl_3_)


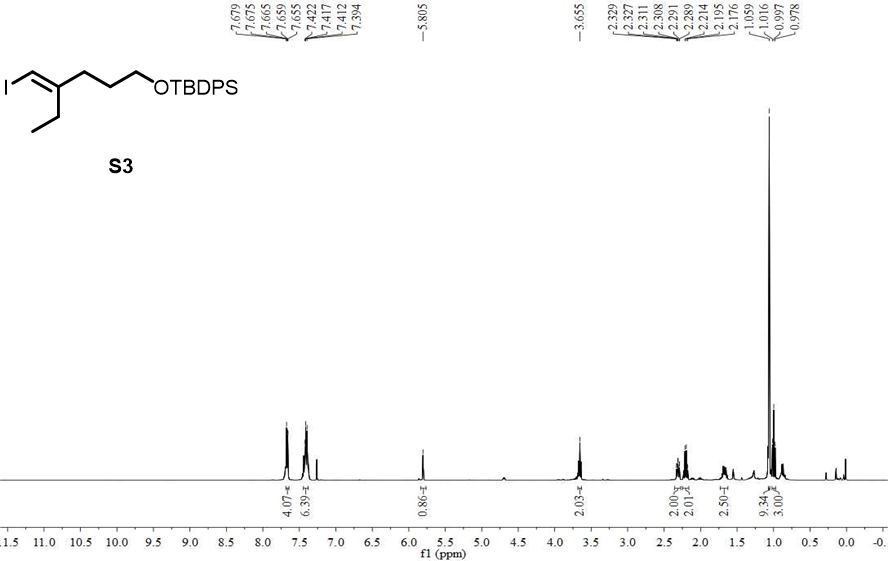


^13^C NMR (101 MHz, CDCl_3_)


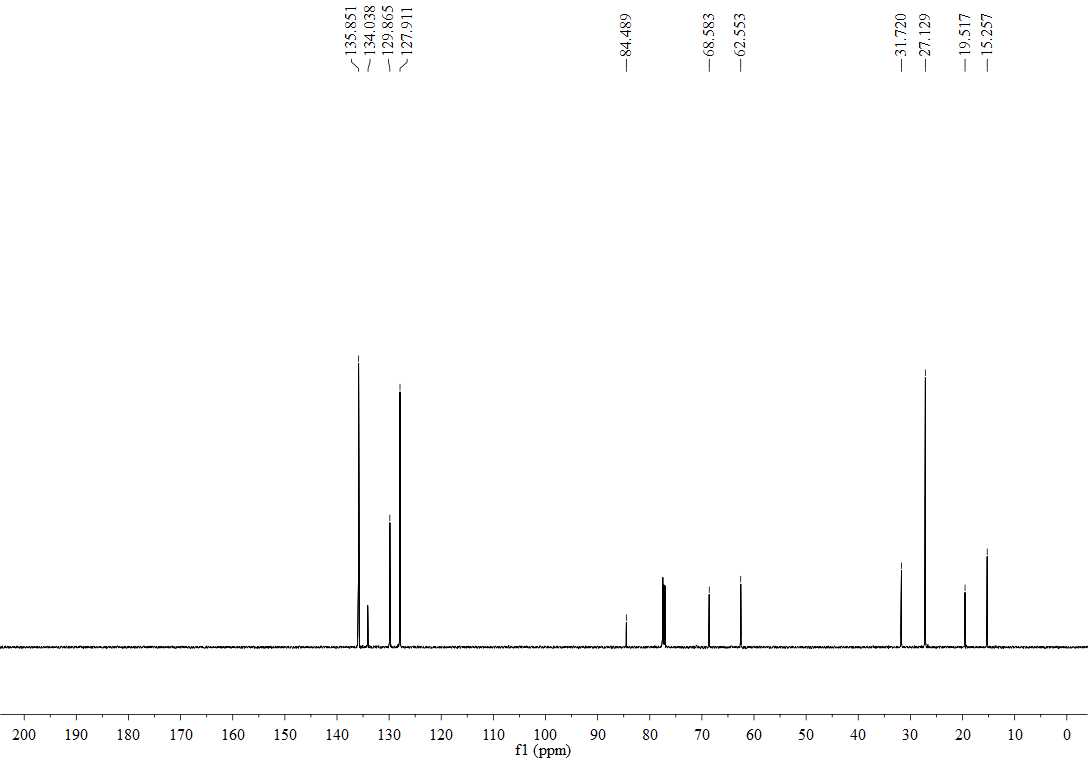


**S4** NMR spectra

^1^H NMR (400 MHz, CDCl_3_)


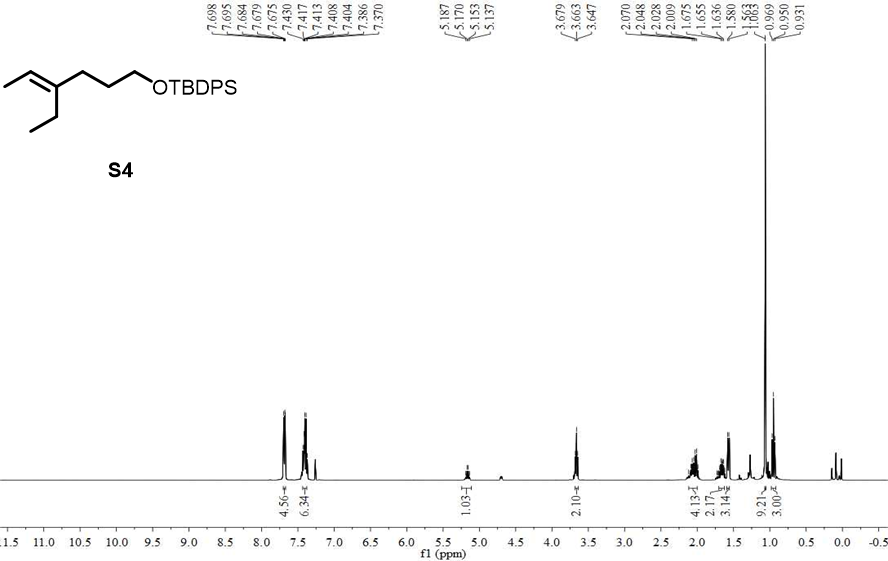


^13^C NMR (101 MHz, CDCl_3_)


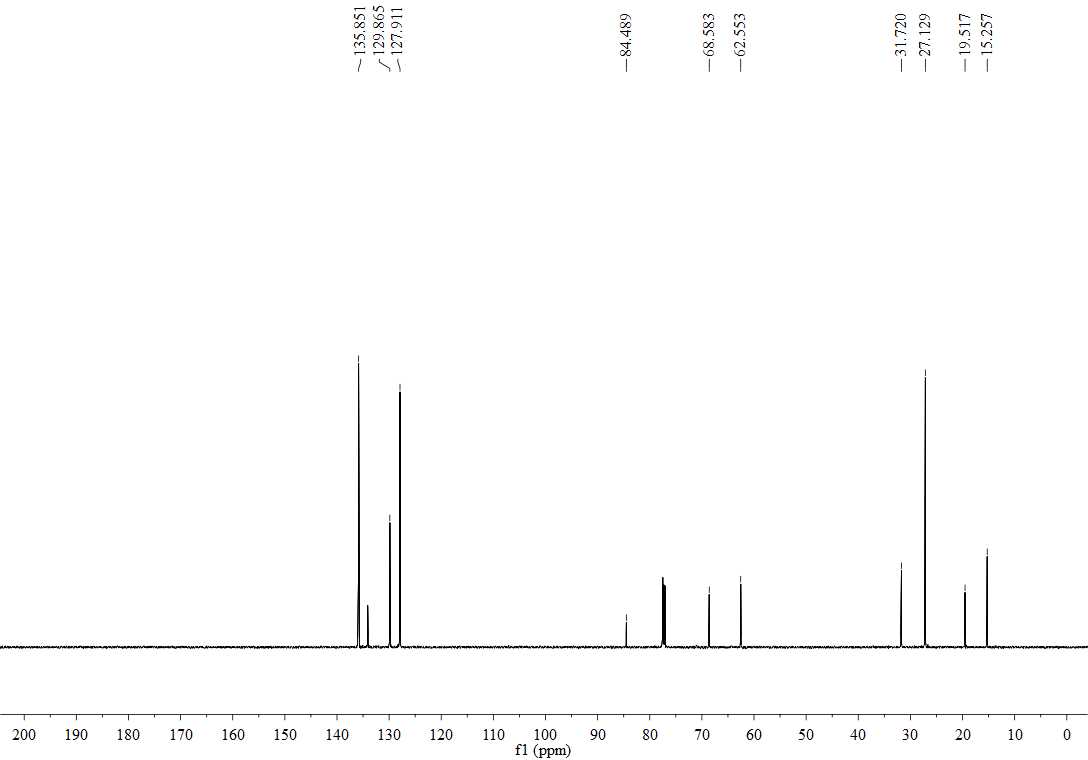


**S5** NMR spectra

^1^H NMR (400 MHz, CDCl_3_)


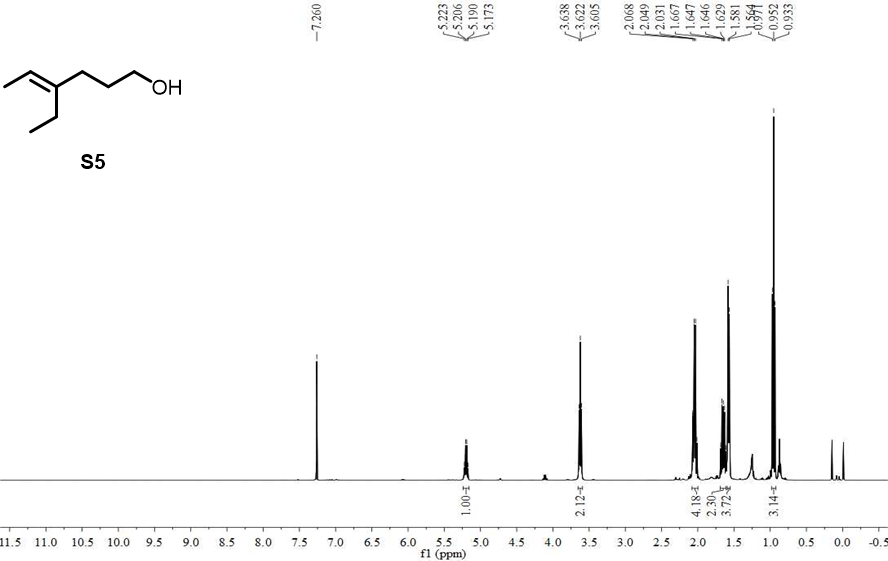


^13^C NMR (101 MHz, CDCl_3_)


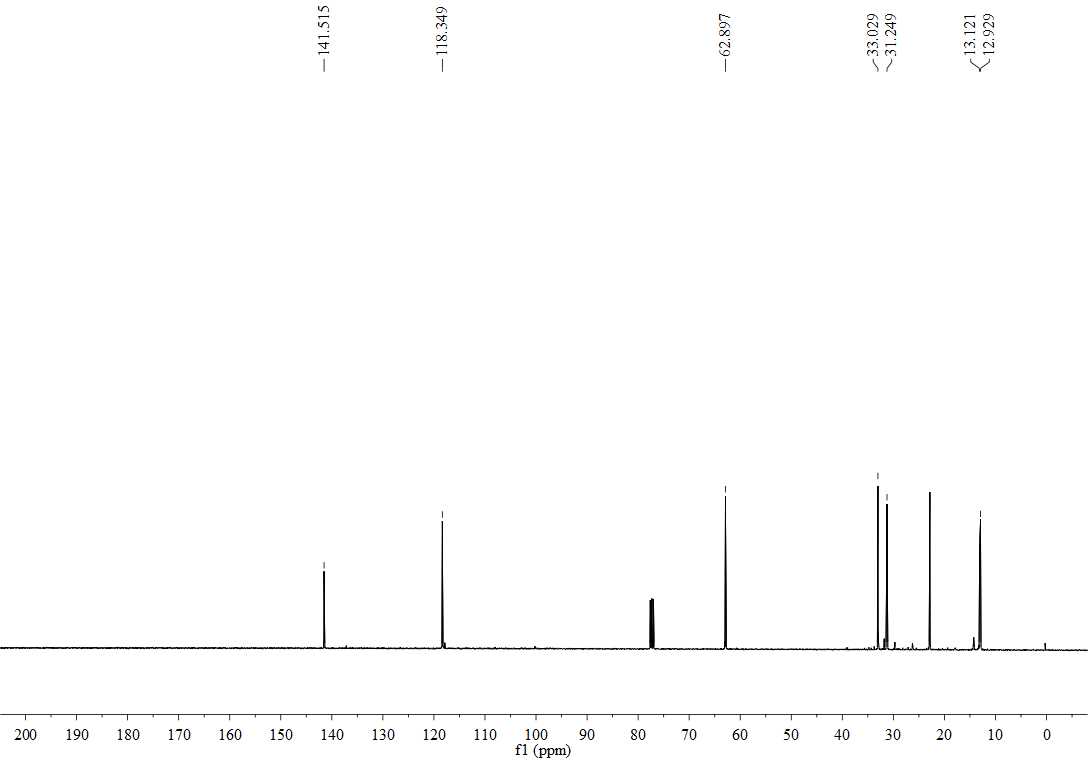


**S6** NMR spectra

^1^H NMR (400 MHz, CDCl_3_)


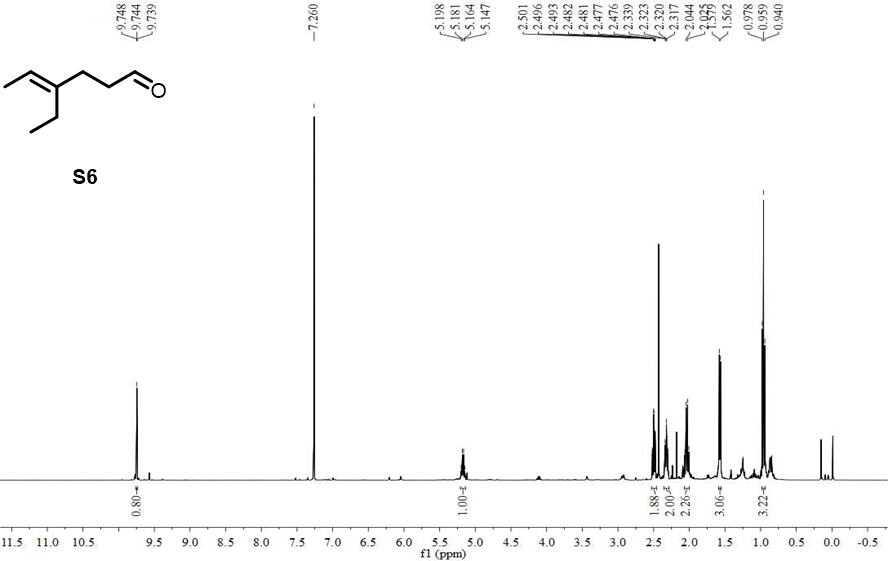


^13^C NMR (101 MHz, CDCl_3_)


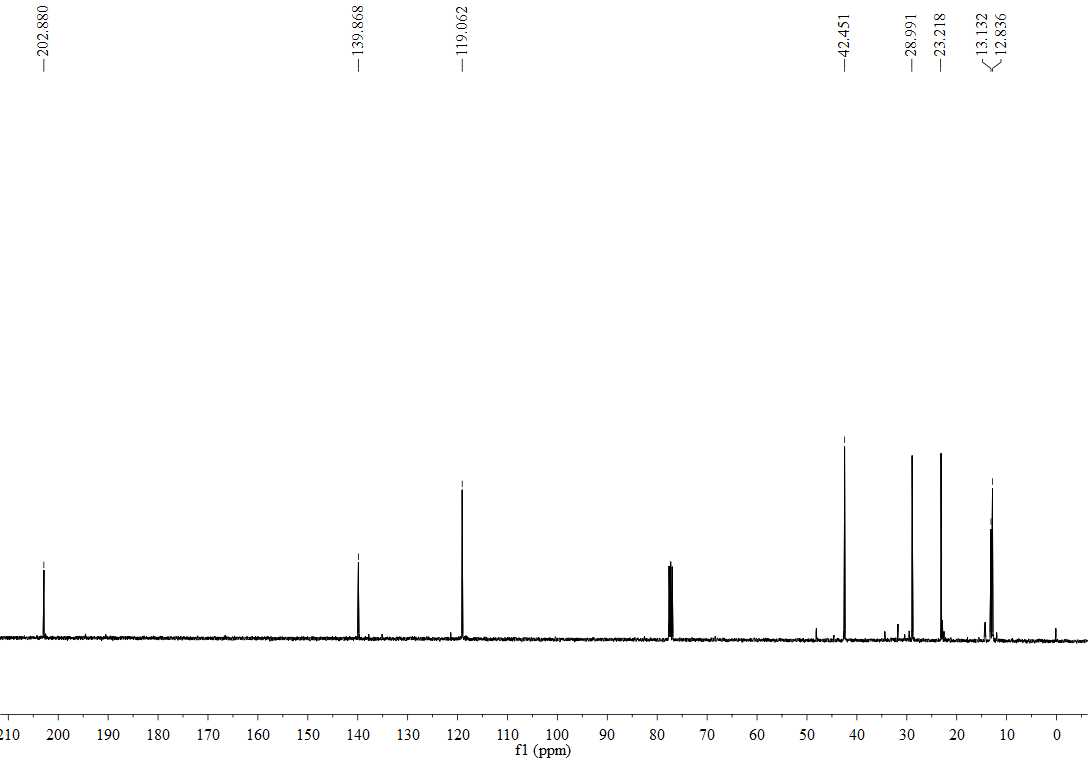


**S7** NMR spectra

^1^H NMR (400 MHz, CDCl_3_)


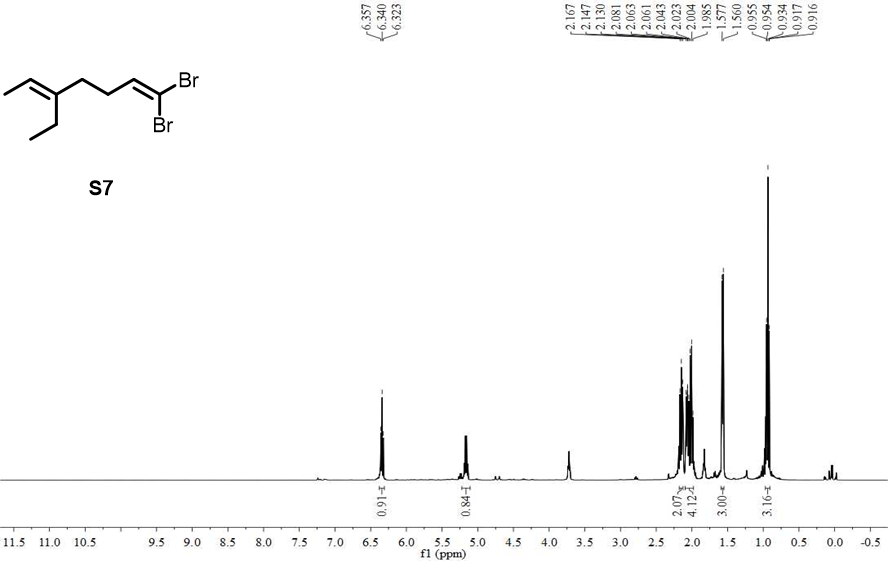


^13^C NMR (101 MHz, CDCl_3_)


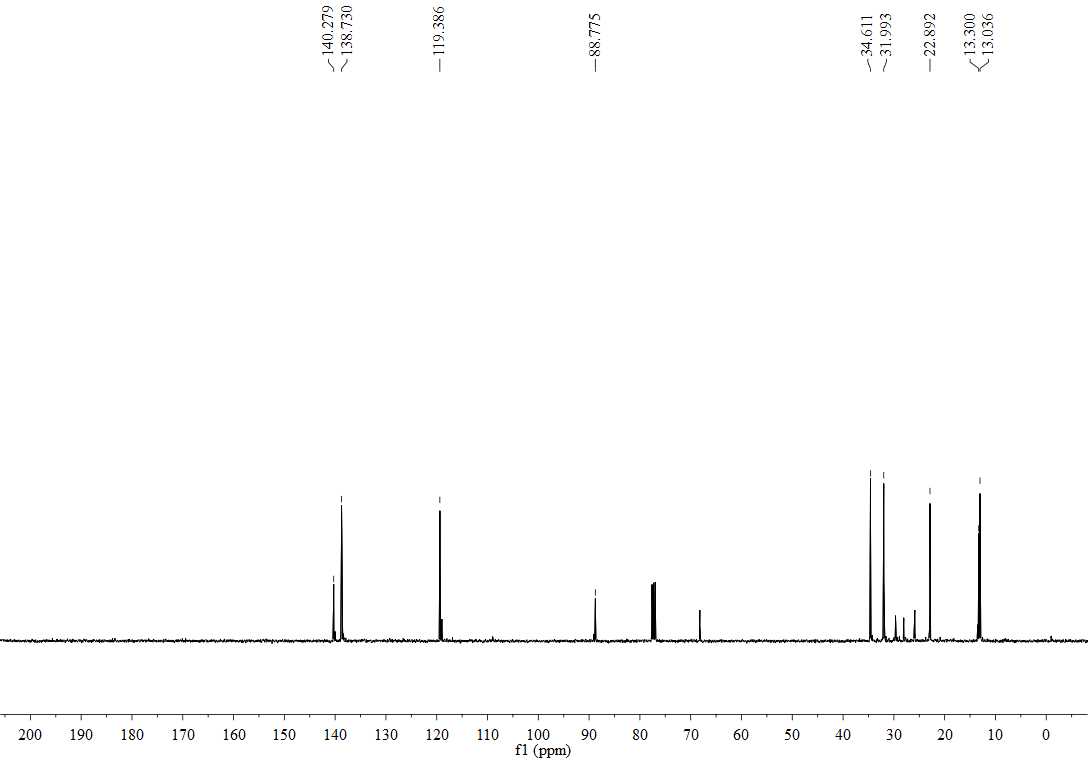


**S8** NMR spectra

^1^H NMR (400 MHz, CDCl_3_)


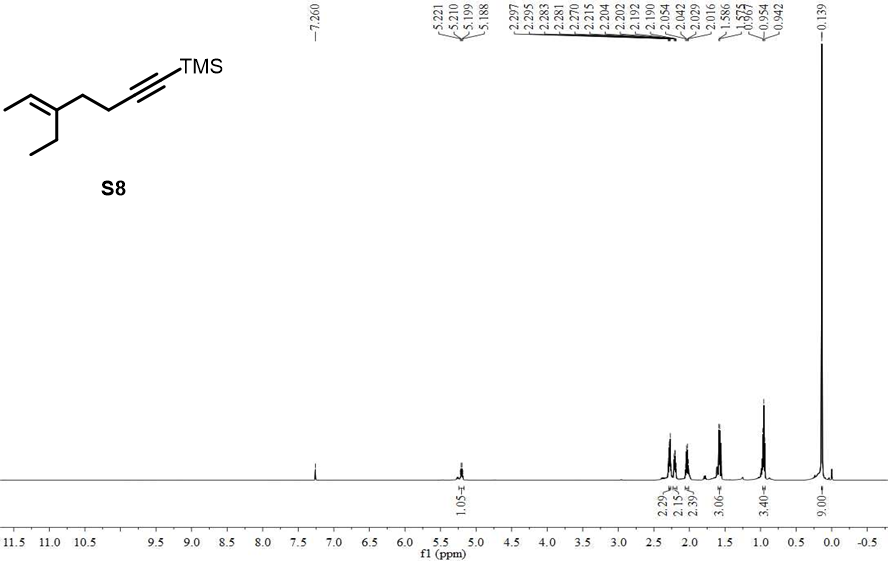


^13^C NMR (101 MHz, CDCl_3_)


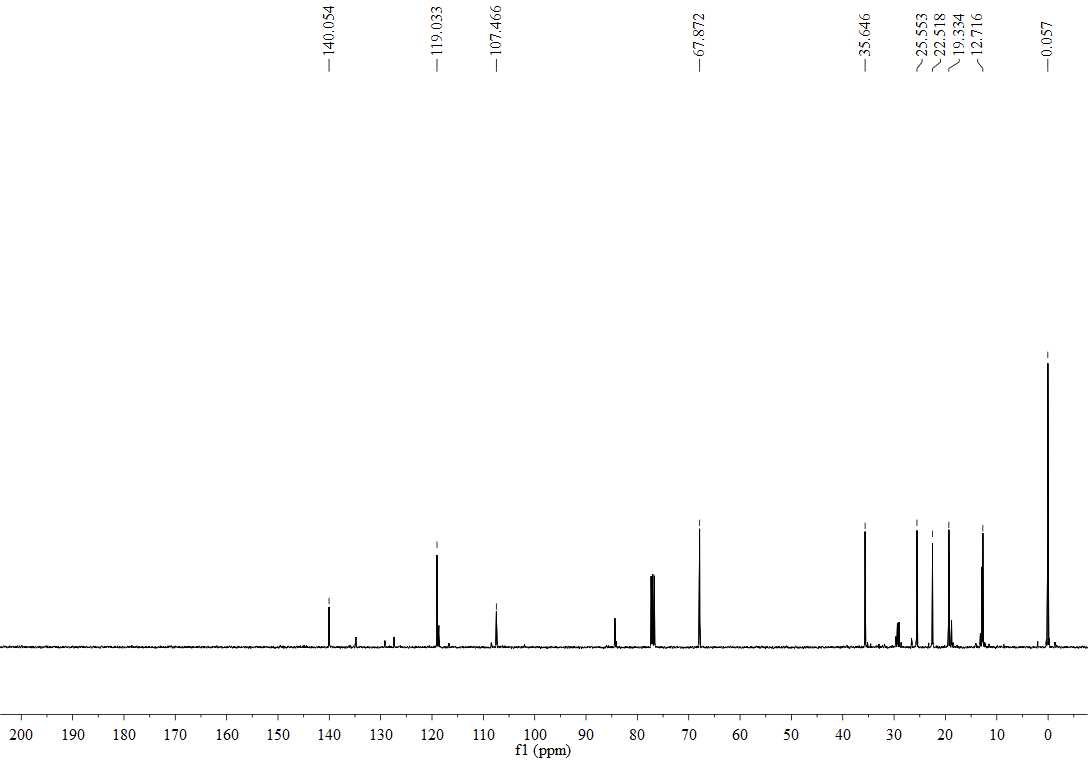


**S9** NMR spectra

^1^H NMR (400 MHz, CDCl_3_)


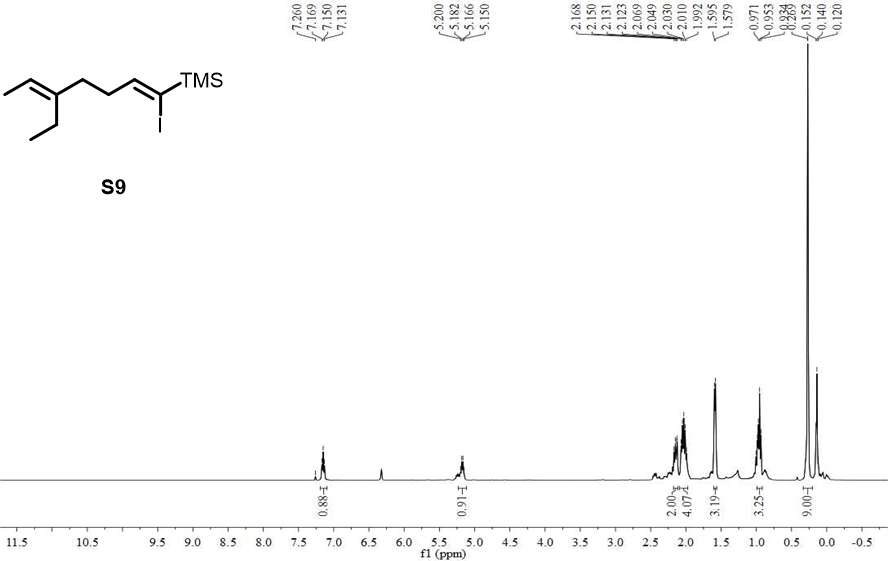


^13^C NMR (101 MHz, CDCl_3_)


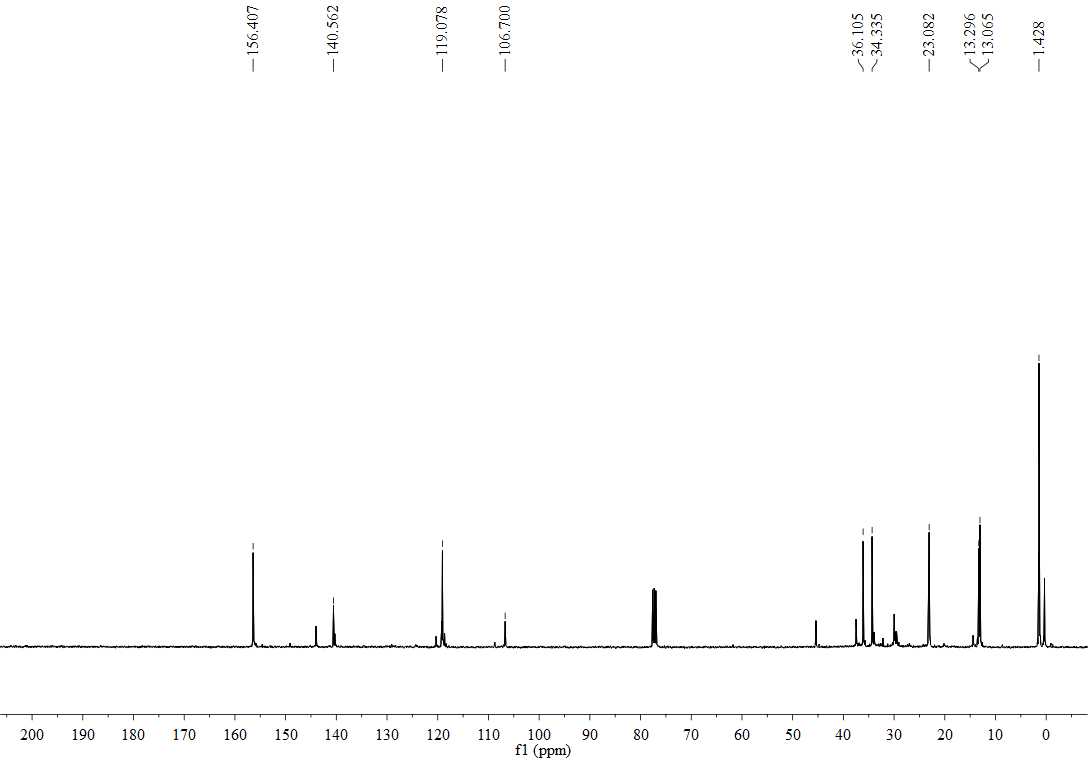


**S10** NMR spectra

^1^H NMR (400 MHz, CDCl_3_)


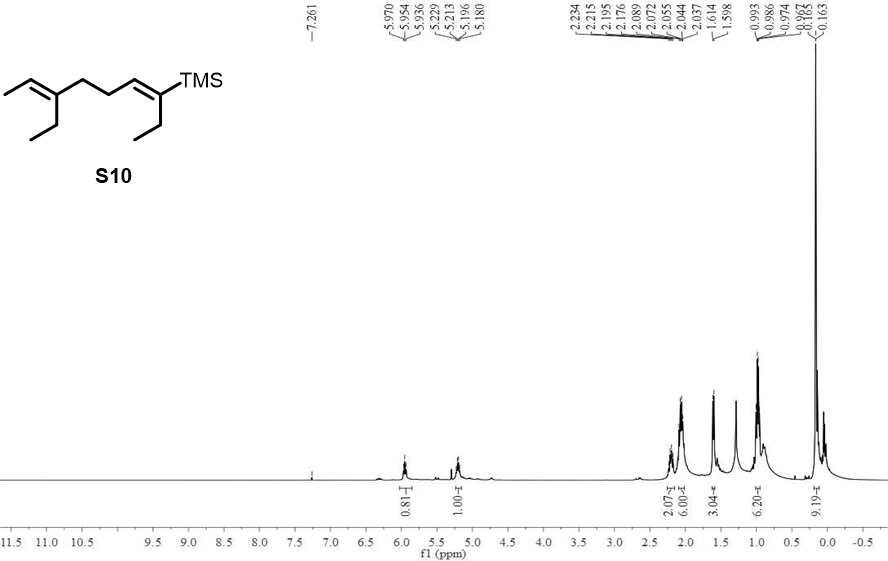


^13^C NMR (101 MHz, CDCl_3_)


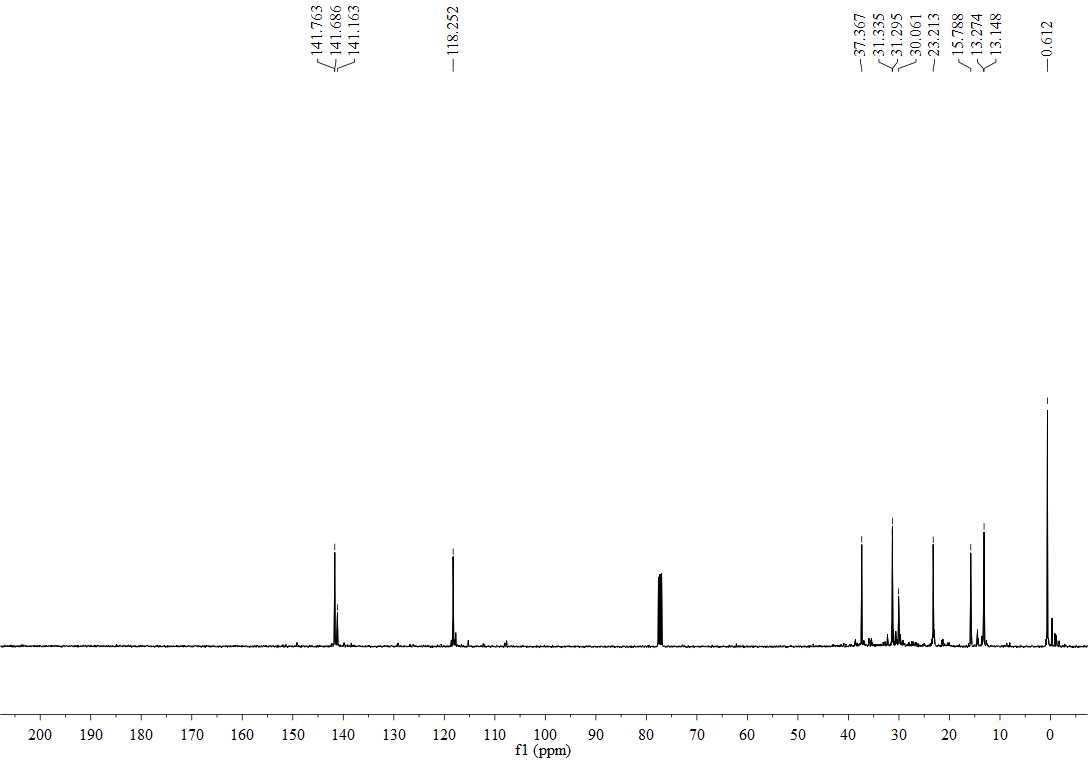


**S11** NMR spectra

^1^H NMR (400 MHz, CDCl_3_)


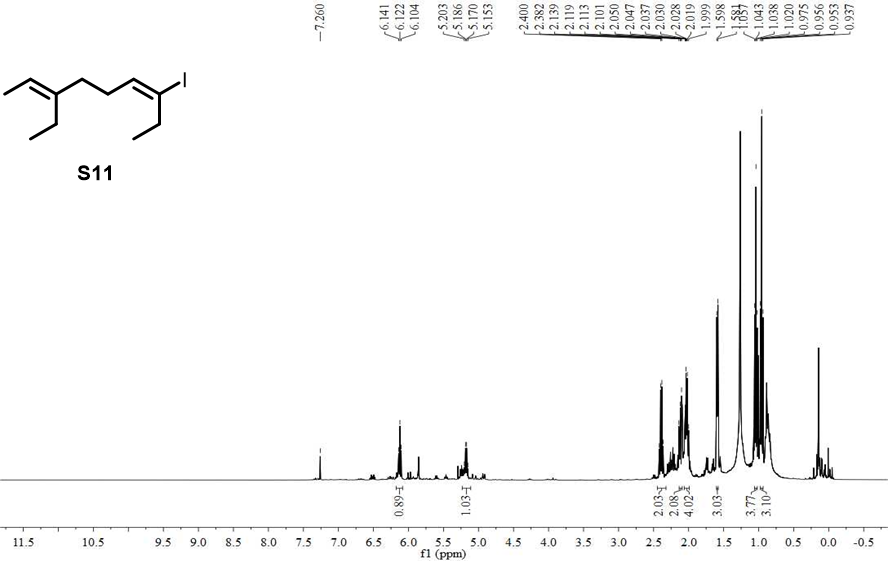


^13^C NMR (101 MHz, CDCl_3_)


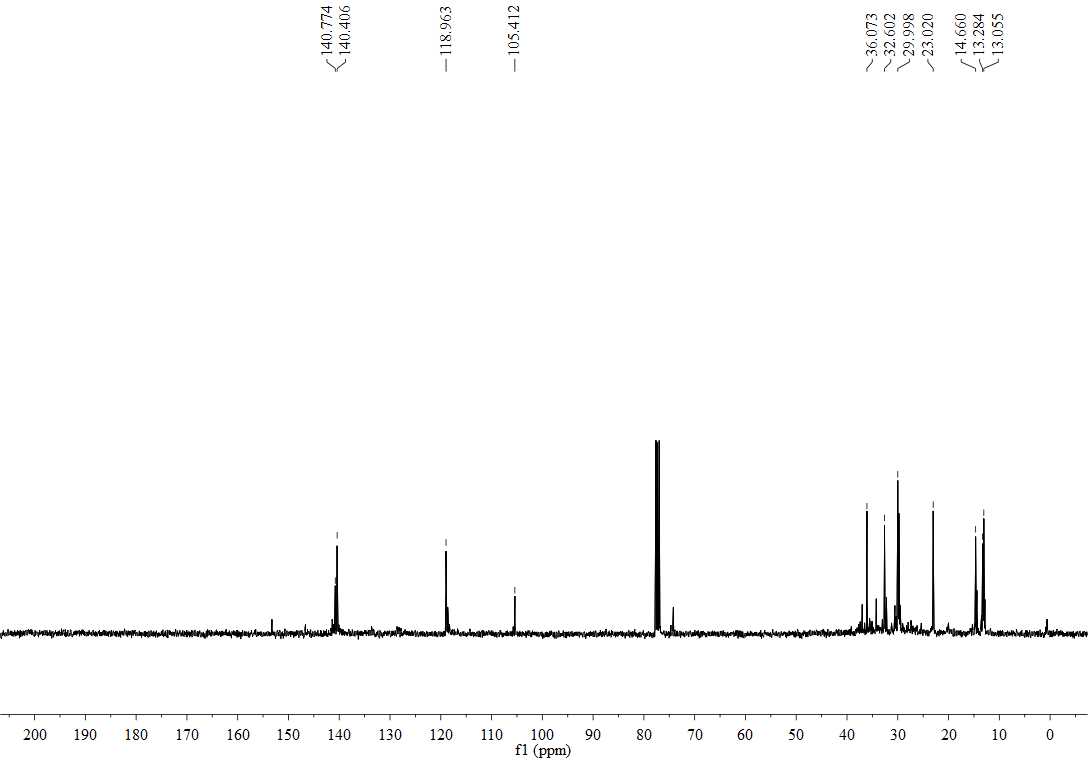


**P1** NMR spectra

^1^H NMR (400 MHz, CDCl_3_)


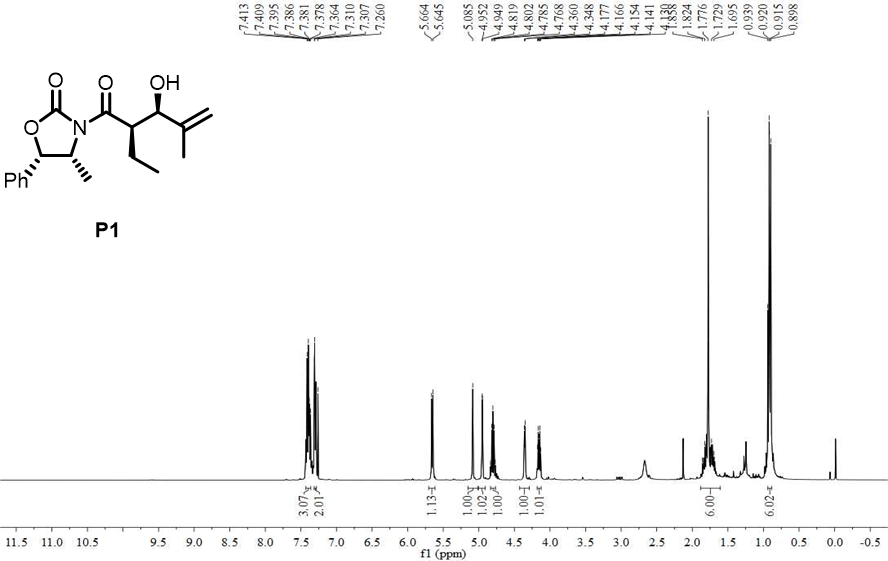


^13^C NMR (101 MHz, CDCl_3_)


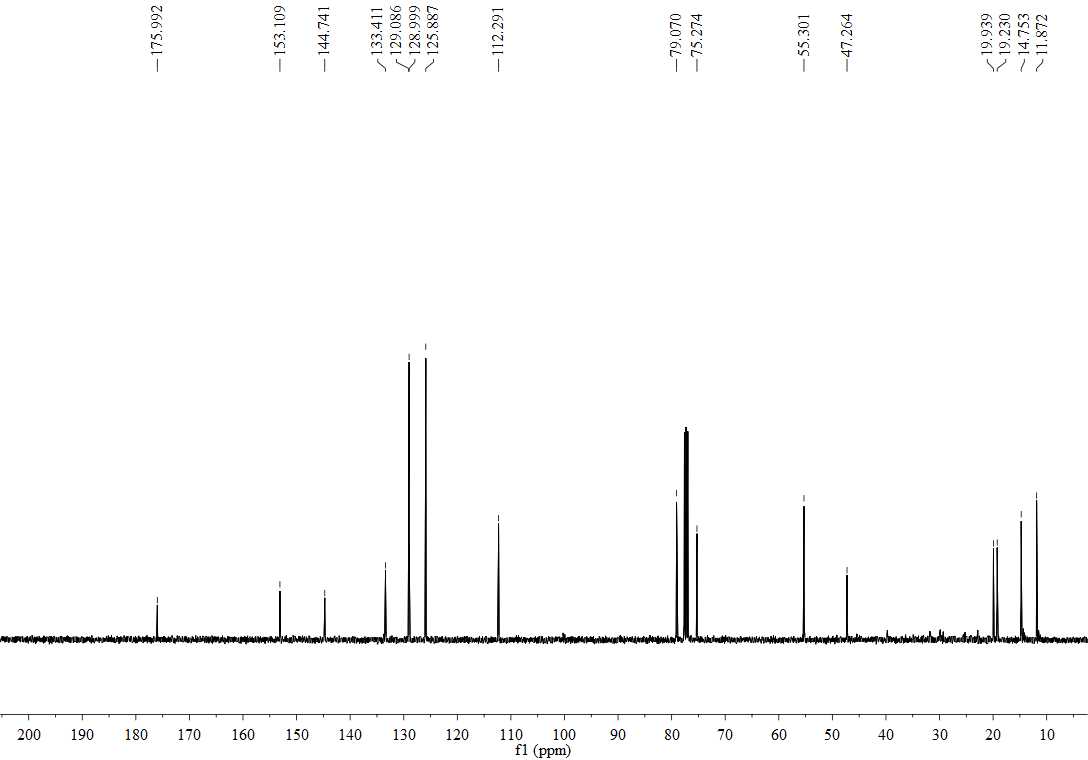


**P2** NMR spectra

^1^H NMR (400 MHz, CDCl_3_)


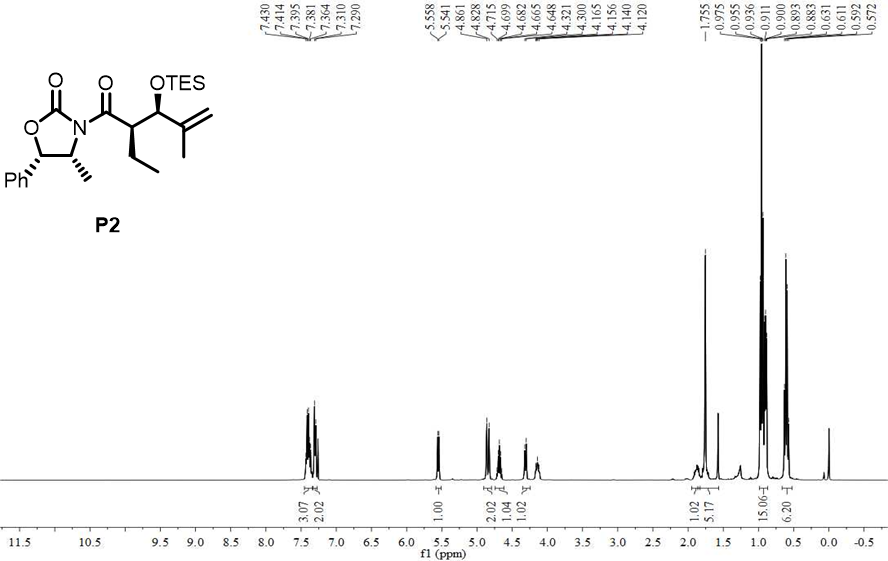


^13^C NMR (101 MHz, CDCl_3_)


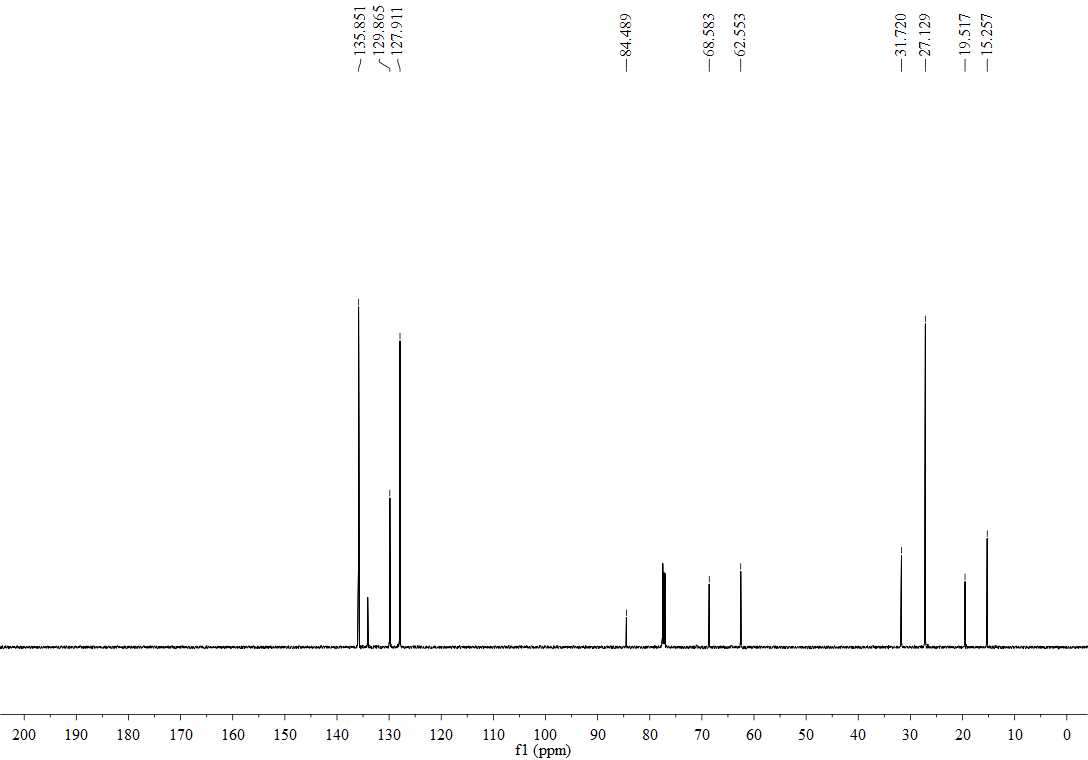


**P3** NMR spectra

^1^H NMR (400 MHz, CDCl_3_)


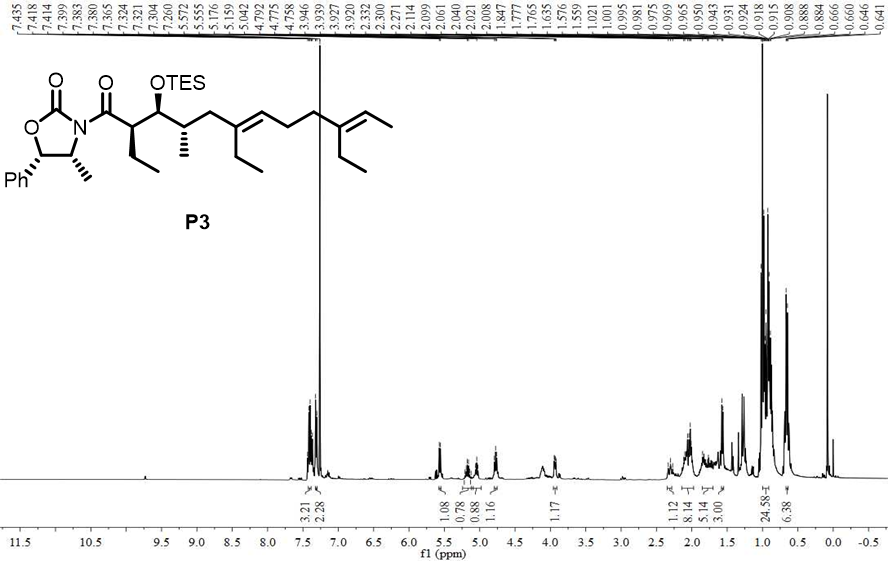


^13^C NMR (101 MHz, CDCl_3_)


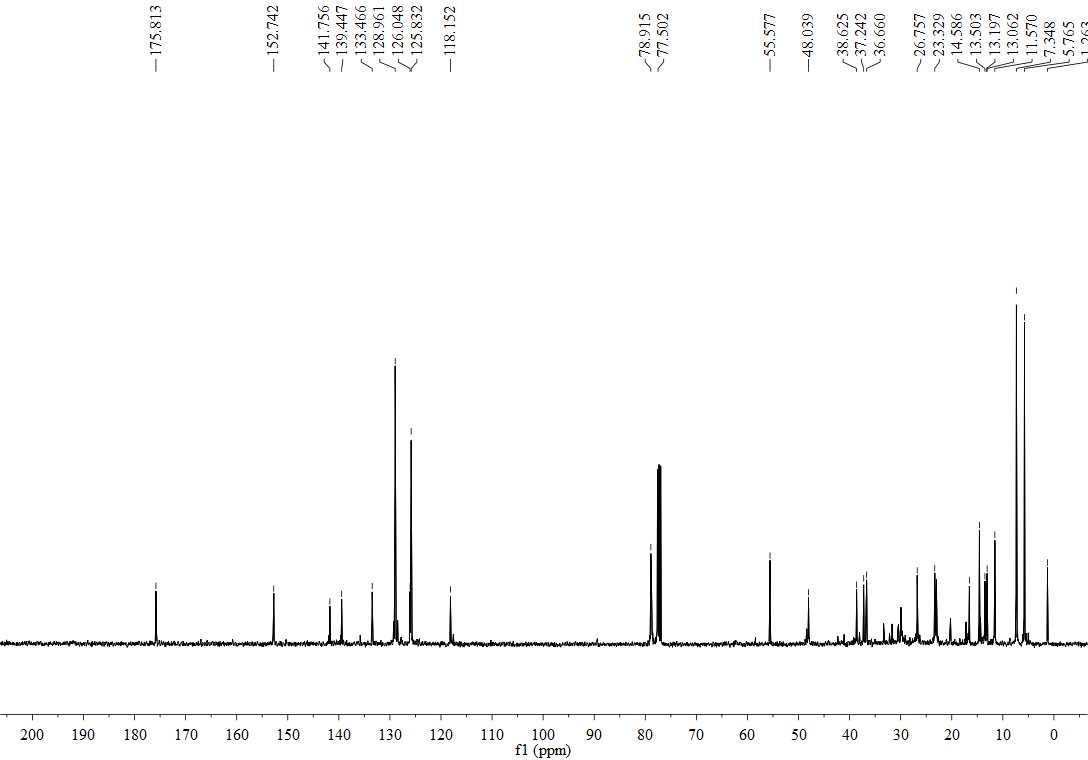


**1** NMR spectra

^1^H NMR (400 MHz, CDCl_3_)


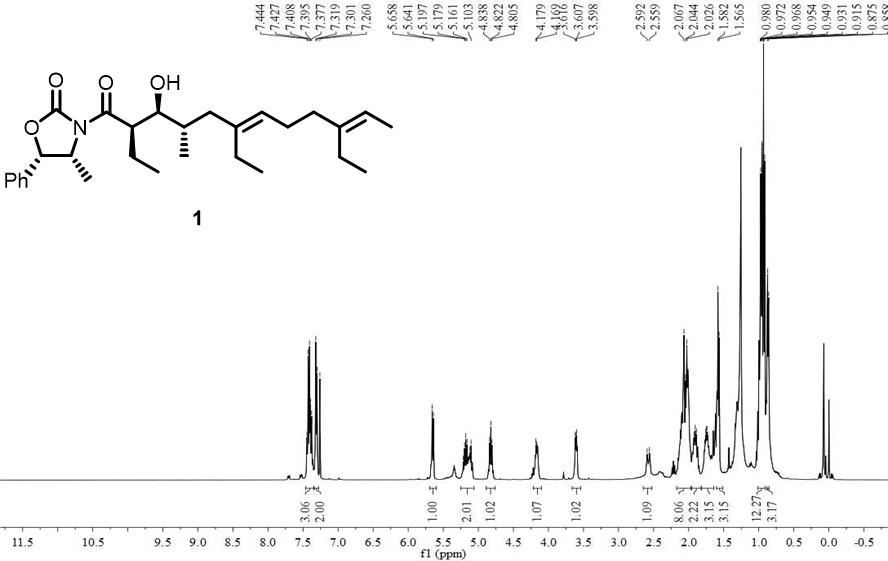


^13^C NMR (101 MHz, CDCl_3_)


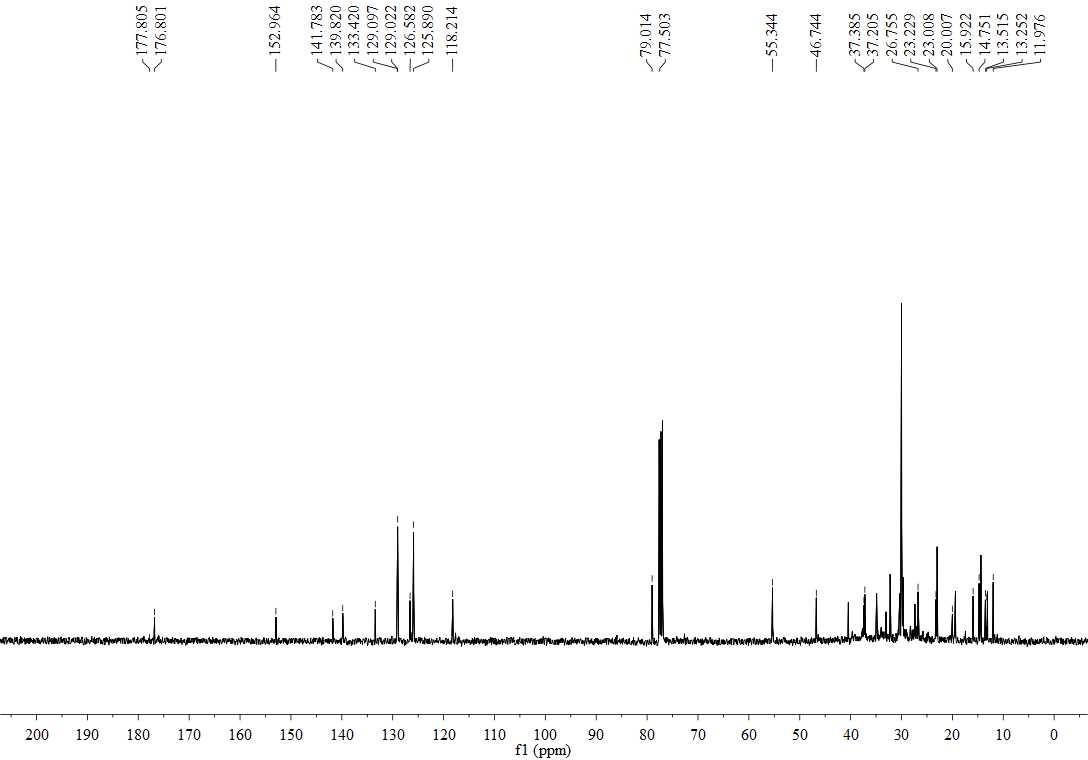


**2** NMR spectra

^1^H NMR (400 MHz, CDCl_3_)

**
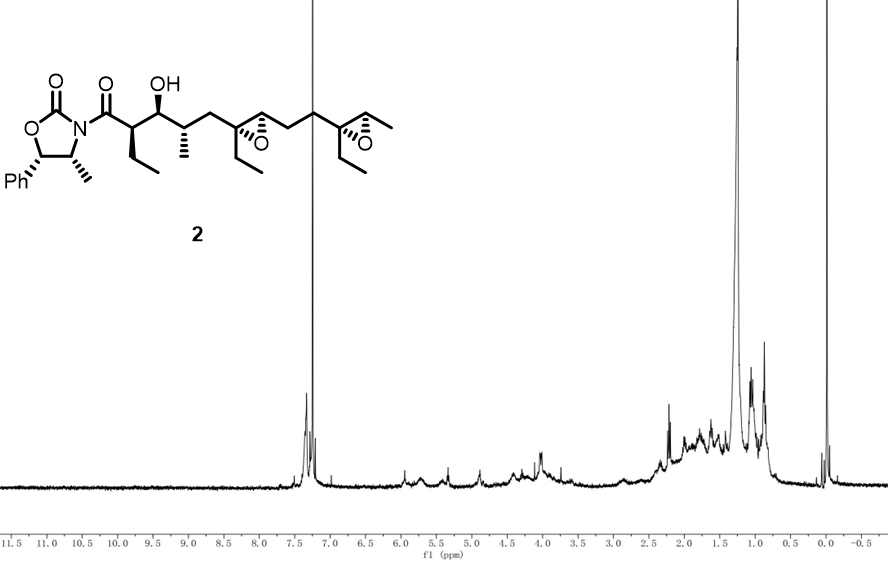
**

**P5** (**5a**, **5b**, **5c**, **5d**) NMR spectra

^1^H NMR (400 MHz, CDCl_3_)


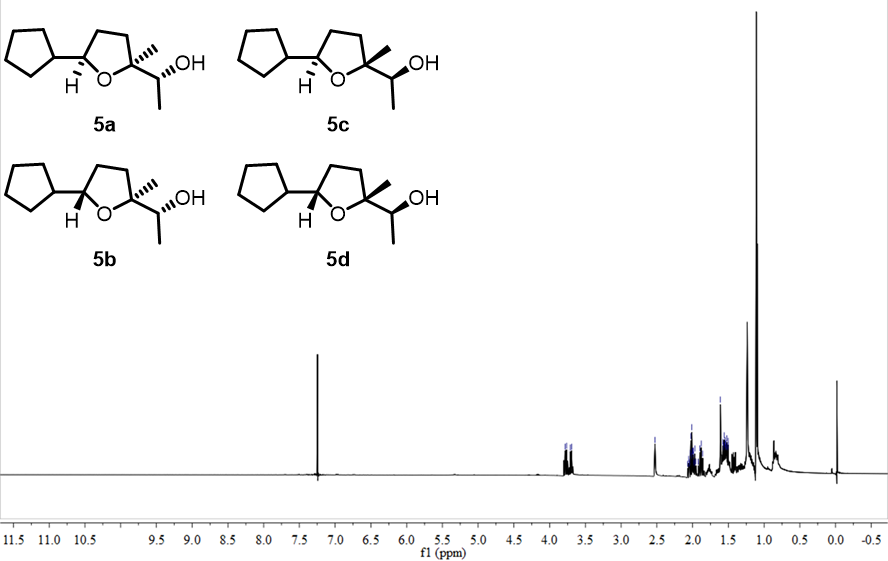


^13^C NMR (101 MHz, CDCl_3_)


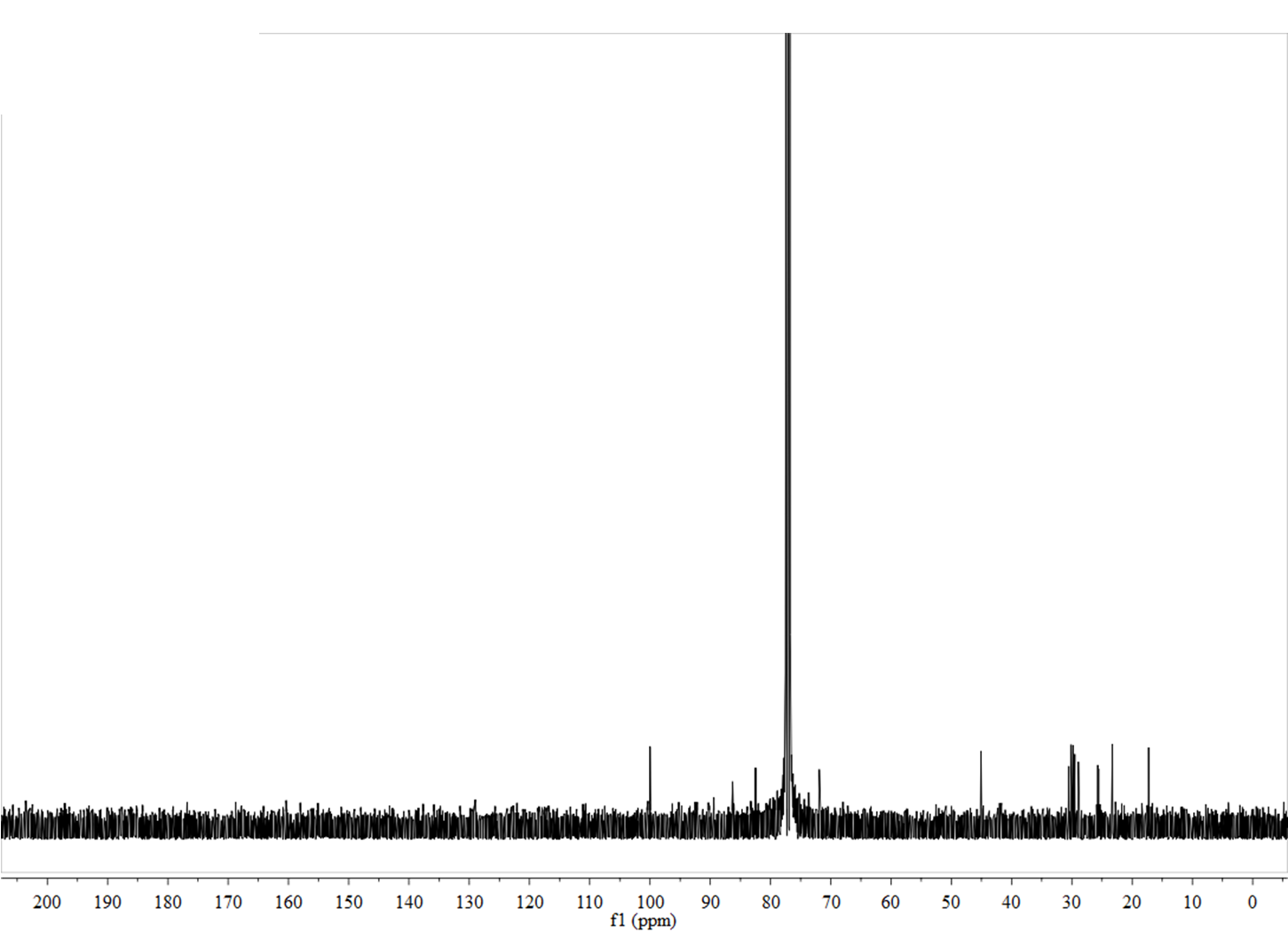


**P6 (5a, 5b)** NMR spectra

^1^H NMR (400 MHz, CDCl_3_)


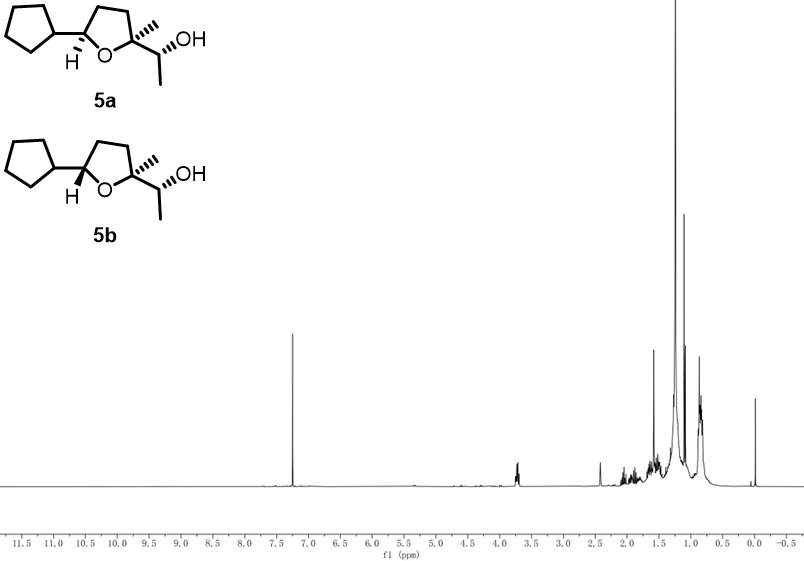


^13^C NMR (101 MHz, CDCl_3_)


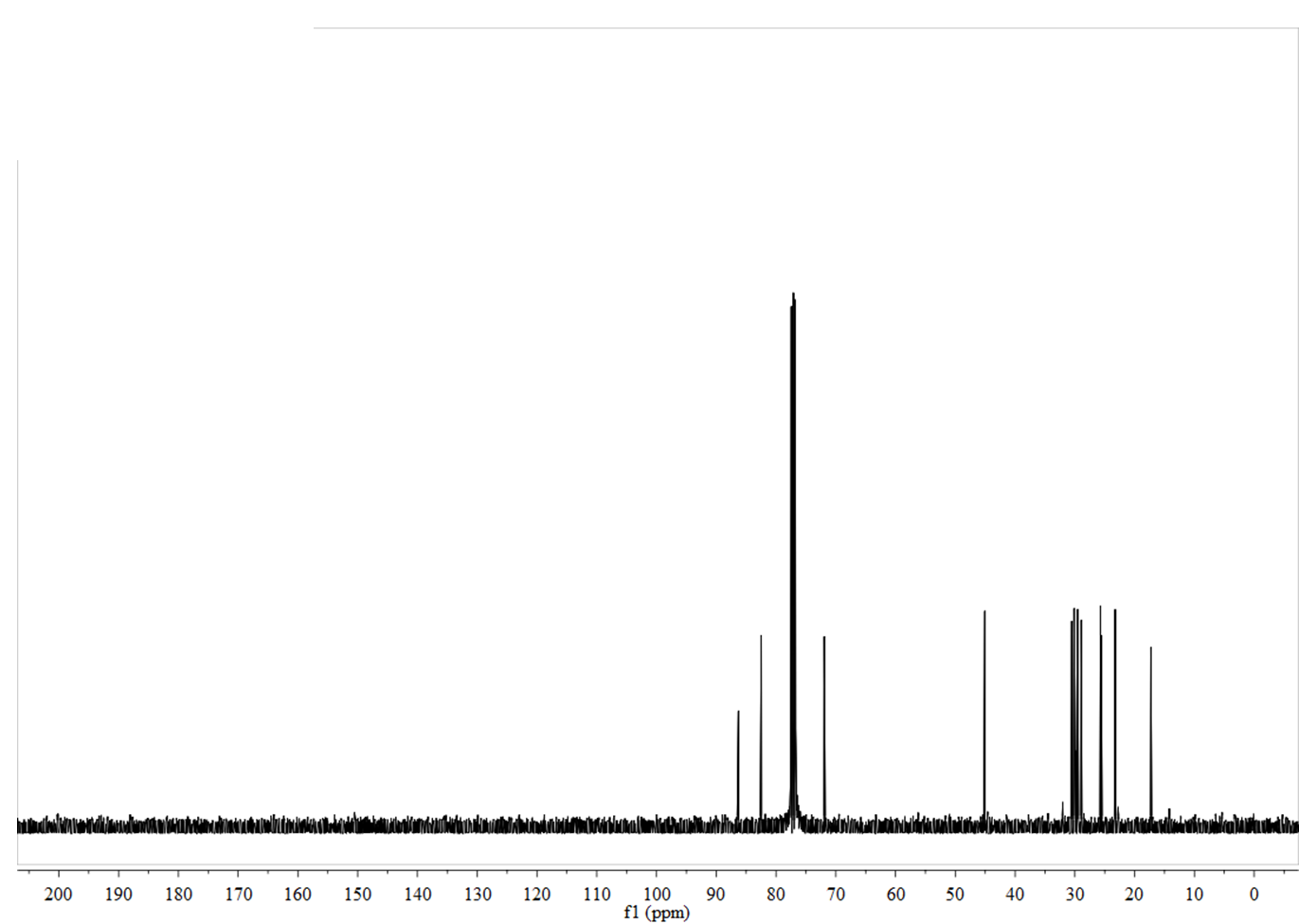


MS data of **2**


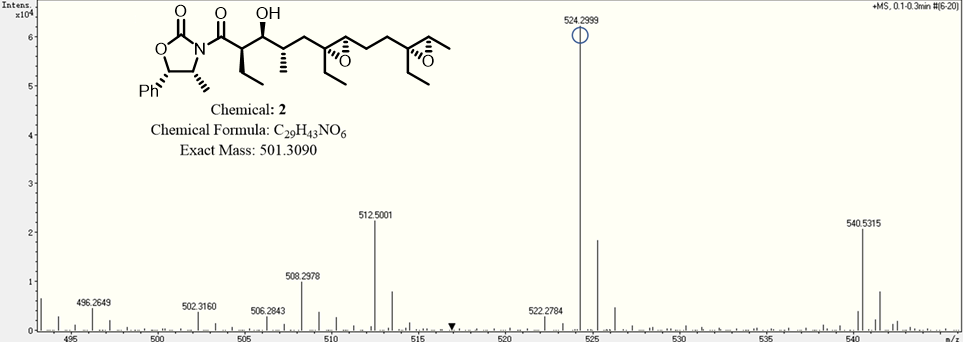


**Supplementary Tables**

**Table S1. Data collection and refinement statistics**

|  | Lsd18-FAD  (PDB ID: 8UP4) | Lsd18-FAD-**1**  (PDB ID: 8XTZ) | Lsd18-FAD-**2**  (PDB ID: 8XU7) |
| --- | --- | --- | --- |
| **Data collection** |  |  |  |
| Space group | *P1* | *P*1 | *P*1 |
| Cell dimensions |  |  |  |
| *a*, *b*, *c* (Å) | 46.35, 61.79, 75.36 | 46.6, 61.8, 75.7 | 47.0, 62.2, 76.3 |
| α, β, γ (°) | 74.64, 81.70, 77.11 | 74.6, 81.8, 77.0 | 74.8, 82.0, 76.8 |
| Wavelength (Å) | 1.0000 | 0.9792 | 0.9792 |
| Resolution (Å) | 1.51 (1.59-1.51) | 1.85 (1.89-1.85) | 2.20 (2.27-2.20) |
| *R*_sym_ (%) | 5.8 (41.6) | 11.6 (21.8) | 12.6 (65.0) |
| I/σ (I) | 11.6 (2.9) | 11.9 (5.1) | 10.7 (3.2) |
| Completeness (%) | 95.9 (94.1) | 97.2 (90.3) | 98.0 (97.3) |
| Redundancy | 3.5 (3.4) | 1.0 (1.0) | 6.9 (6.8) |
| CC_1/2_ | 0.998 (0.871) | 0.982 (0.954) | 0.995 (0.332) |
|  |  |  |  |
| **Refinement statistics** |  |  |  |
| Resolution limit (Å) | 36.75-1.51 (1.56-1.51) | 25.33-1.85 (1.92-1.85) | 19.61-2.20 (2.28-2.20) |
| No. of reflections | 117,632 (16,922) | 65,614 (6,174) | 40,336 (3,985) |
| *R*_work_/*R*_free_ (%) | 14.0/17.8 | 16.1/20.2 | 22.7/27.1 |
| Number of atoms |  |  |  |
| Protein | 7,070 | 6,895 | 6,977 |
| Ligand | 108 | 146 | 159 |
| Ion | 2 | 2 | 2 |
| Water | 899 | 1037 | 277 |
| B-factors (Å^2^) |  |  |  |
| Protein | 23.07 | 19.74 | 32.70 |
| Ligand | 17.50 | 19.20 | 34.53 |
| Ion | 23.81 | 21.81 | 36.00 |
| Water | 35.87 | 32.54 | 33.69 |
| **R.m.s. deviations** |  |  |  |
| Bond lengths (Å) | 0.009 | 0.006 | 0.003 |
| Bond angles (°) | 1.01 | 0.81 | 0.60 |

Values in parentheses are for the highest resolution shell.

*R*_sym_ = Σ | *I*avg – *I*i | / Σ *I*i, where *I*i is the observed intensity and *I*avg is the average intensity of observations of symmetry-related reflections.

*R*_work_ = Σ | *F*p – *F*p(calc.) | / Σ *F*p, where *F*p and Fp(calc.) are observed and calculated structure factors; *R*_free_ is calculated with 5% of the data.

**Table S2. Primers for site-directed mutagenesis**

All the mutagenesis experiments were done through overlap PCR. Sequencing was done for all the mutants to confirm the accuracy of the cloning.

| Primer | Sequences of primer (5'→3') |
| --- | --- |
| Lsd18-F-*Nde*I | GGAATTCCATATGACGAACACGCGCTC |
| Lsd18-R-EcoRI | CCGGAATTCTTAAGCGGTAACACCAG |
| I72A-F | CATGCGCACGCTCTGTGGTCTGGCGGTG |
| I72A-R | GACCACAGAGCGTGCGCATGACGAGCTTGC |
| Y218A-F | AGCGGTATGGTCGCTGCTACCCGTC |
| Y218A-R | AGACGGGTAGCAGCGACCATACCGC |
| Y218F-F | GTATGGTCTTTGCTACCCGTCTGTTTCGTG |
| Y218F-R | CGGGTAGCAAAGACCATACCGCTATCAAC |
| Y218S-F | GTATGGTCTCTGCTACCCGTCTGTTTCGTG |
| Y218S-R | CGGGTAGCAGAGACCATACCGCTATCAAC |
| V252A-F | GTTGTAATGCAGCGCTGATGCCGATTG |
| V252A-R | AATCGGCATCAGCGCTGCATTACAACC |
| V342A-F | CGTTTAATCCGGCGTACGGCCATGG |
| V342A-R | GCCATGGCCGTACGCCGGATTAAAC |

**Supplementary Figures**


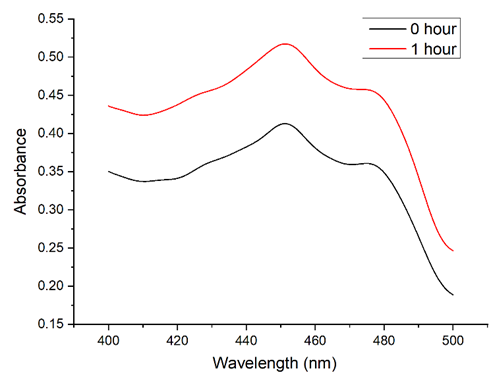


**Figure S1*.*** UV-Vis spectrum of Lsd18 immediately after purification (black) and one hour after incubation (red). Reaction mixture contained 30 µM Lsd18, 80 µM farnesyl acetate as substrate, 2mM NADH, 2 mM NADPH, 10% methanol, 300 mM NaCl, 5% glycerol and 50 mM Tris-HCl pH 8.0.

**
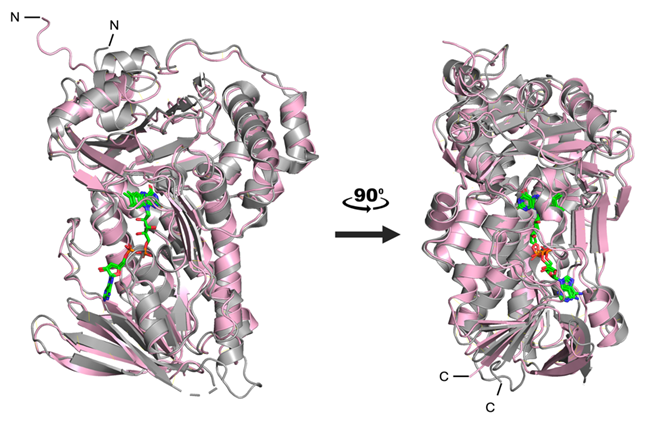
**

**Figure S2*.*** Structural superposition of Lsd18 and MonCI. X-ray crystal structure of Lsd18 (PDB ID: 8UP4) is drawn in pink and the X-ray crystal structure of MonCI (PDB ID: 8T3P) is drawn in grey. Root mean square deviation of the superimposed C_α_ positions is 1.2 Å.


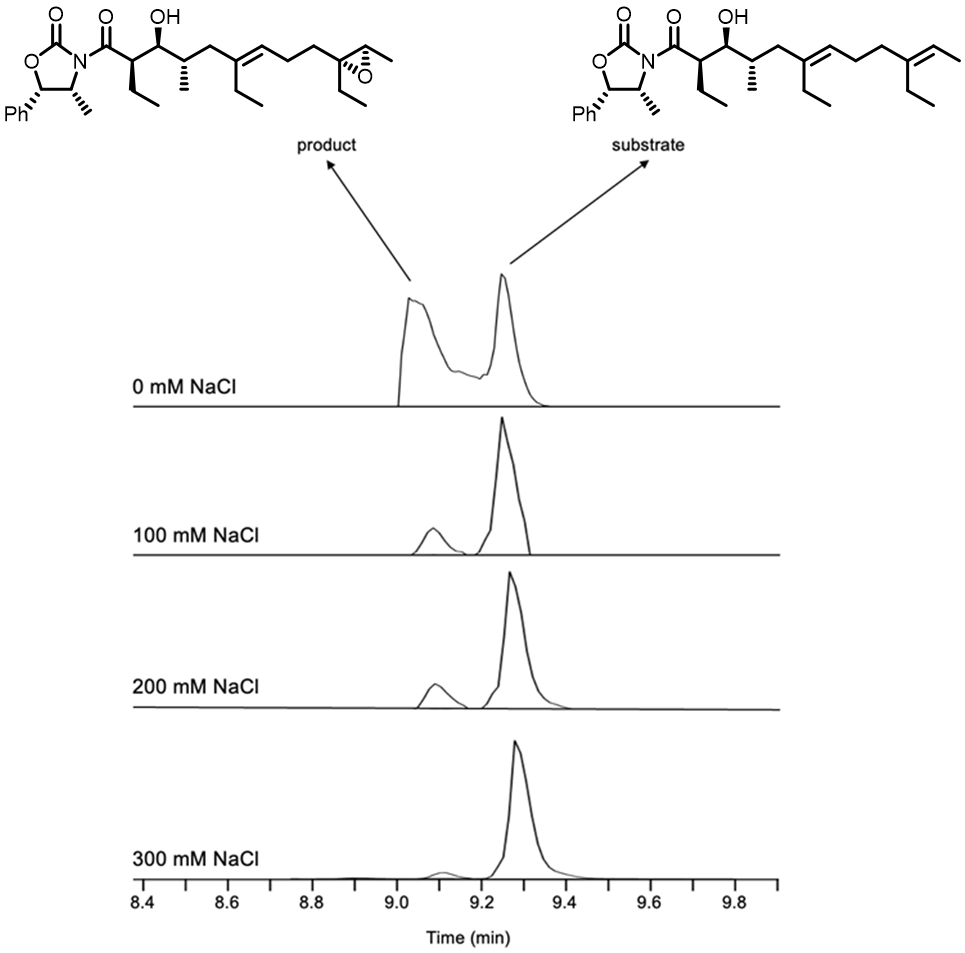


**Figure S3*.*** Effect of chloride ion on Lsd18 activity. Reactions were carried out in buffer containing 100 mM sodium phosphate at pH 8.0, 80 μM substrate, 1 mM NADH, 1 mM NADPH, 80 μM FAD, 15 μM Lsd18, 15 μM Fre, 30% methanol, 5% glycerol, and 0 to 300 mM NaCl. The reaction mixture was incubated at 30 °C for 1 hour, followed by extraction with an equal volume of ethyl acetate. Substrate and product were detected using liquid chromatography-mass spectrometry.


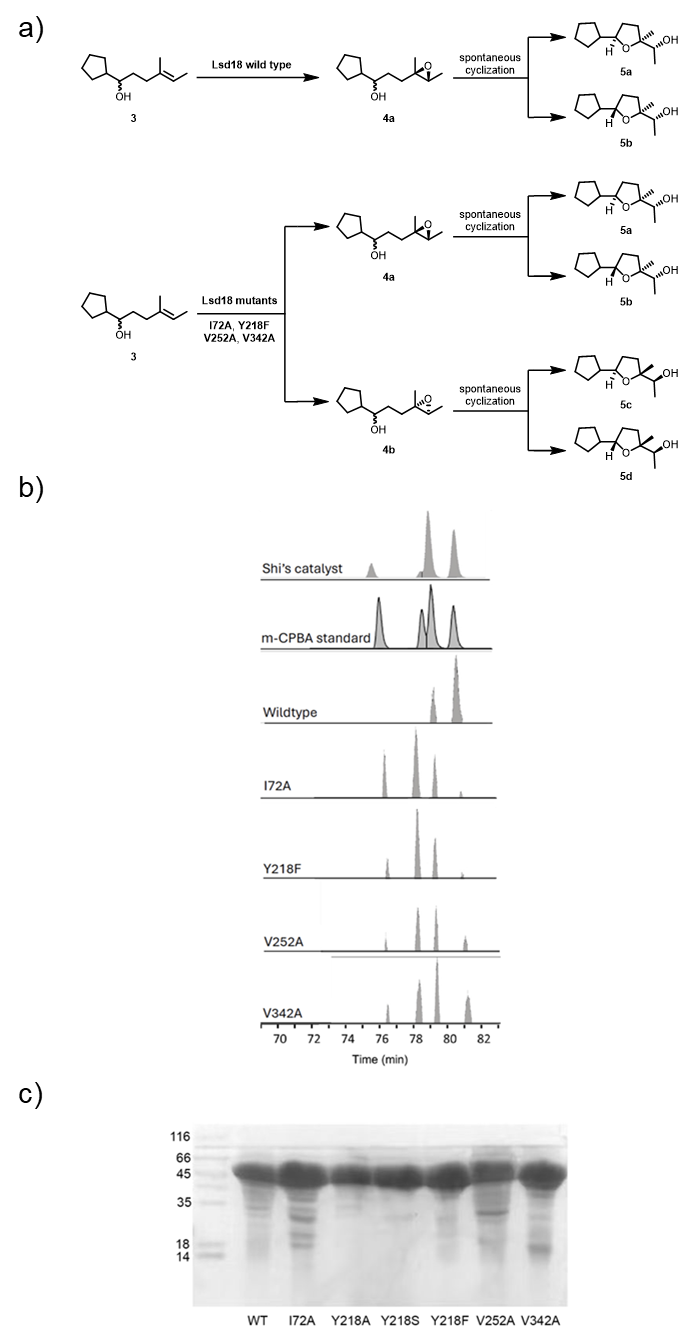


**Figure S4.** SDS-PAGE of recombinant wild-type and mutant Lsd18 proteins.


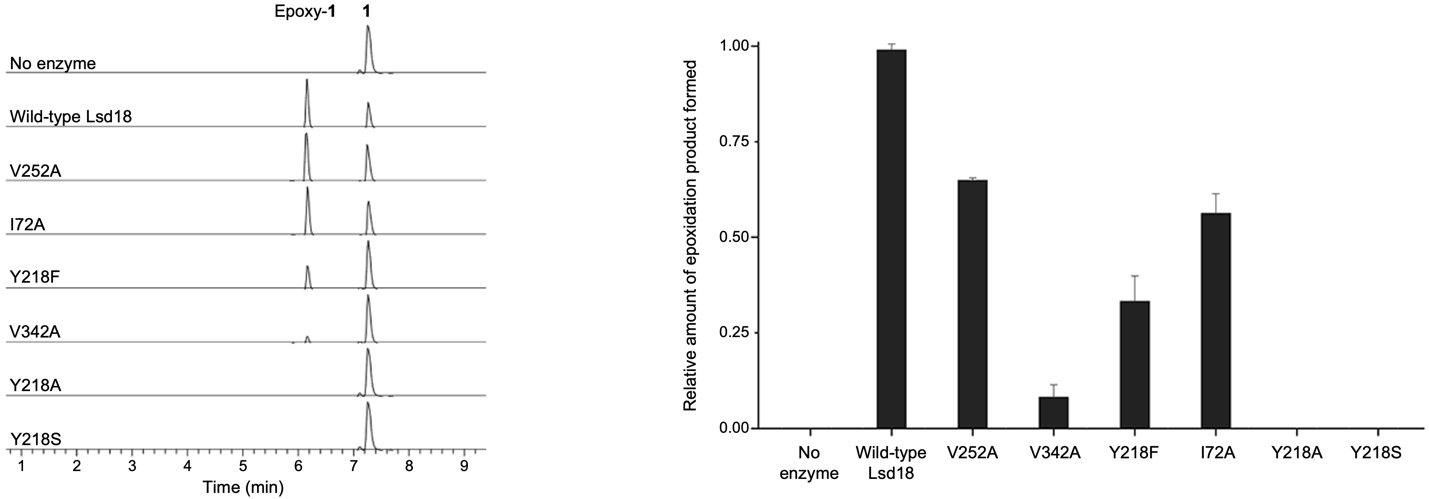


**Figure S5.** Relative catalytic efficiency of the wild-type, V252A, I72A, Y218F, V342A, Y218A, and Y218S Lsd18 proteins. The amount of epoxy-**1** formed was quantified by calculating the area of the product peak in the liquid chromatography profile.


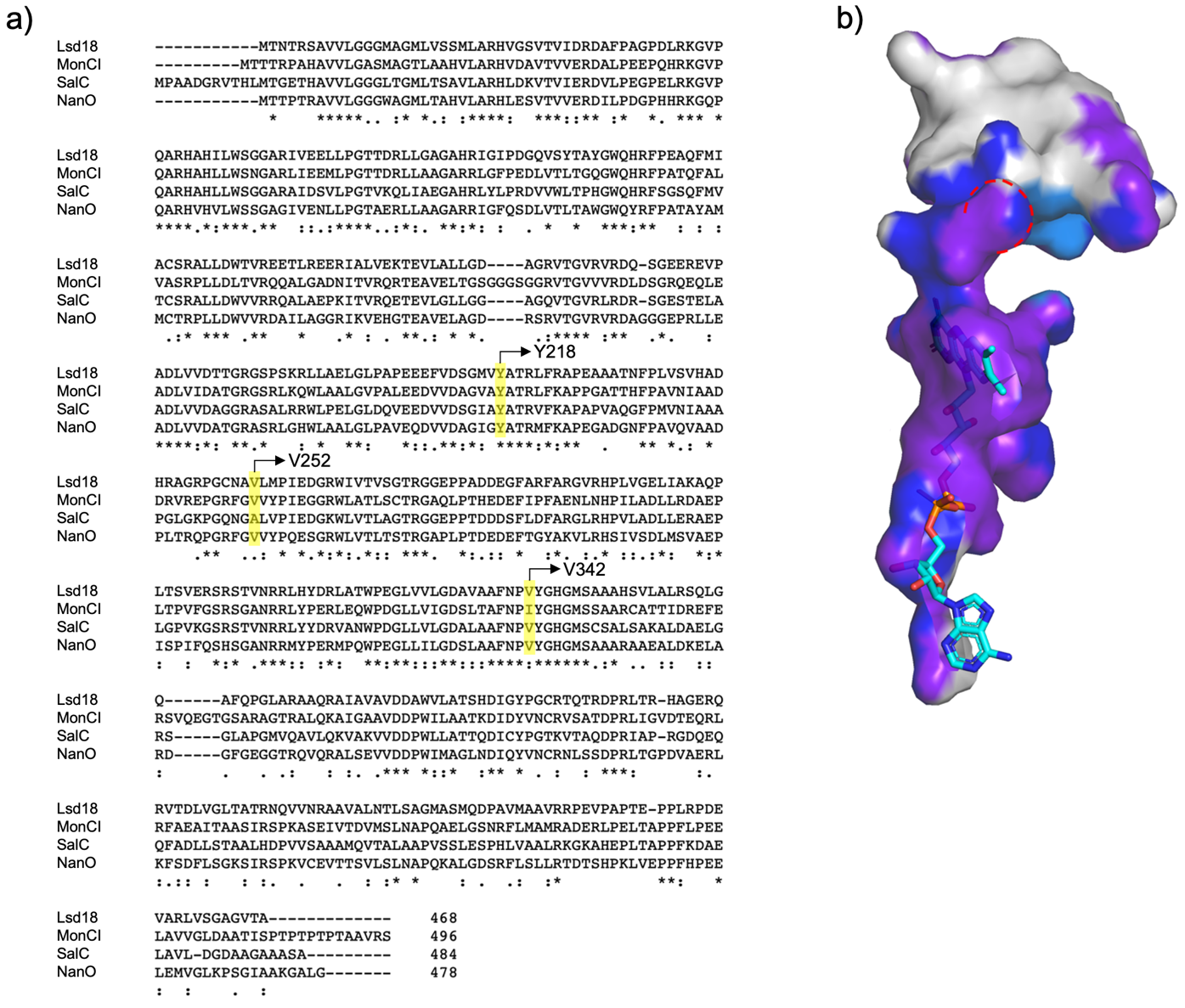


**Figure S6.** a) Sequence comparison of monooxygenases involved in polyether biosynthesis. Lsd18, MonCI, SalC, and NanO are involved in the biosynthesis of lasalocid A, monensin, salinomycin, and nanchangmycin, respectively. In Lsd18, Y218, V252, and V342 form the subpocket that binds the ethyl substituent of **1**. b) The FAD and substrate binding pocket of Lsd18. The broken red line indicates the approximate outline of the subpocket. Surfaces colored in purple are contributed by absolutely conserved residues among Lsd18, MonCI, SalC, and NanO. Surfaces colored in blue are contributed by highly conserved residues.
